# Vitamin B12 and Folic Acid Improve Gross Motor and Problem-Solving Skills in Young North Indian Children: A Randomized Placebo-Controlled Trial

**DOI:** 10.1371/journal.pone.0129915

**Published:** 2015-06-22

**Authors:** Ingrid Kvestad, Sunita Taneja, Tivendra Kumar, Mari Hysing, Helga Refsum, Chittaranjan S. Yajnik, Nita Bhandari, Tor A. Strand, Folate and Vitamin B12 Study Group

**Affiliations:** 1 Department of Biological and Medical Psychology, Faculty of Psychology, University of Bergen, Bergen, Norway; 2 Regional Centre for Child and Youth Mental Health and Child Welfare, West, Uni Research Health, Bergen, Norway; 3 Society for Applied Studies, New Delhi, India; 4 Society for Essential Health Action and Training, New Delhi, India; 5 Institute of Basic Medical Sciences, Department of Nutrition, University of Oslo, Oslo, Norway; 6 Department of Pharmacology, University of Oxford, Oxford, United Kingdom; 7 The Diabetes Unit, King Edward Memorial Hospital, Maharashtra, India; 8 Centre for International Health, University of Bergen, Bergen, Norway; 9 Division of Medical Services, Innlandet Hospital Trust, Lillehammer, Norway; University of Ottawa, CANADA

## Abstract

**Objectives:**

Deficiencies of vitamin B12 and folate are associated with delayed development and neurological manifestations. The objective of this study was to measure the effect of daily supplementation of vitamin B12 and/or folic acid on development in young North Indian children.

**Methods:**

In a randomized, double blind trial, children aged six to 30 months, received supplement with placebo or vitamin B12 and/or folic acid for six months. Children were allocated in a 1:1:1:1 ratio in a factorial design and in blocks of 16. We measured development in 422 children by the Ages and Stages Questionnaire 3rd ed. at the end of the intervention.

**Results:**

Compared to placebo, children who received both vitamin B12 and folic acid had 0.45 (95% CI 0.19, 0.73) and 0.28 (95% CI 0.02, 0.54) higher SD-units in the domains of gross motor and problem solving functioning, respectively. The effect was highest in susceptible subgroups consisting of stunted children, those with high plasma homocysteine (> 10 μmol/L) or in those who were younger than 24 at end study. With the exception of a significant improvement on gross motor scores by vitamin B12 alone, supplementation of either vitamin alone had no effect on any of the outcomes.

**Conclusion:**

Our findings suggest that supplementation of vitamin B12 and folic acid benefit development in North Indian Children.

**Trial Registration:**

ClinicalTrials.gov NCT00717730

## Introduction

Poor vitamin B12 status is common among young children in many low- to middle-income countries (LMIC) [1–5]. Deficiency in vitamin B12 has been associated with decreased cognitive performance among elderly [6], and case studies in infants show that severe vitamin B12 deficiency can dramatically affect the developing brain [3, 7]. Observational studies in children have reported associations between vitamin B12 deficiency and neurodevelopment [8–10]. For instance, in a cohort study in North Indian children, marginal vitamin B12 status was associated with lower scores on the mental development index of the Bayley Scales of Infant and Toddler Development (Bayley) 2nd ed. [9]. Moreover, six weeks old Norwegian infants with evidence of poor vitamin B12 status had substantially improved motor development one month after a vitamin B12 injection [11].

Poor folate status may also occur in some populations in LMIC, including India [1, 4]. The biochemical and metabolic effects of vitamin B12 and folate are closely related. Deficiency of either vitamin results in elevation of plasma total homocysteine (tHcy) [12, 13], and the consequences for neurodevelopment are similar [10]. In addition to the co-occurrence and possible synergistic effects of micronutrients on development, several relevant factors such as growth, infections and early life psychosocial factors are associated with developmental status in children [14]. Hence randomized placebo-controlled trials (RCT) are called for to clarify the effect of vitamin B12 and/or folate deficiency on early child development [10].

The objective of the current study was to measure the effect of two recommended daily allowances of vitamin B12 and/or folic acid for six months on neurodevelopment. In a RCT in young North Indian children, we compared scores of the different developmental domains of the Ages and Stages Questionnaire 3rd. ed. (ASQ-3) (communication, gross motor, fine motor, problem-solving and personal social) between a placebo group and three intervention groups receiving vitamin B12 and/or folic acid for six months.

## Materials and Methods

### Participants and study setting

The children (n = 422) included in this study participated in a RCT (n = 1000) on the effect of vitamin B12 and/or folic acid supplementation on childhood infections and growth in New Delhi, India [15]. The trial was first registered at www.clinicaltrials.gov as NCT00717730 in July, 2008, and at www.ctri.nic.in as CTRI/2010/091/001090 in August, 2010. The authors confirm that all ongoing and related trials for this intervention are registered.

The study children aged six to 30 months were recruited from low to middle socioeconomic class families living in the Tigri and Dakshinpuri area in New Delhi with a total population of about 300,000, and randomized in blocks of 16. The last 440 enrollments were requested to participate in this developmental assessment sub study. Enrollment was from November 2010 to March 2011, and the developmental assessment was conducted from May through September 2011. The study was approved by the ethics committees of the Society for Essential Health Action and Training (India), Society for Applied Studies (India), Christian Medical College, Vellore (India), and the Norwegian Regional Committee for Medical and Health Research Ethics (REK VEST), July 2008.

### Enrollment and randomization

A door-to-door survey was conducted to identify eligible children in all households in the area. A physician and field supervisors screened the children for ongoing illnesses and measured the hemoglobin levels. Cases of anemia were treated with oral iron as per national guidelines. Availability of informed consent and no plans to move away over the next six months were considered for enrollment. We excluded children with severe acute malnutrition (weight-for-height z-scores < -3), and severe anemia (hemoglobin <7 g/dL), and they were referred for treatment according to national guidelines. Children already using folic acid and/or vitamin B12 supplements were not included in the study. We also excluded children that participated in other trials and children with illnesses requiring hospitalization. With no screening of developmental delays prior to enrollment, only children with known developmental disabilities were excluded. Only one child from each household was recruited for the study. Written informed consent was obtained prior to enrollment from the caregiver on behalf of the children. In case of non-literates, an impartial witness witnessed the consent. All witnesses were registered in a list. The ethics committees approved the consent procedure. Demographic information was collected at enrollment.

Using a factorial design, children were randomized in a 1:1:1:1 ratio in blocks of 16 to one of four treatment groups: placebo, vitamin B12 only, folic acid only, and vitamin B12 and folic acid (in the following, referred to as vitamin B12/folic acid). The randomization was stratified into infants (<12 months) and older children (≥ 12 months) by assigning blocks to either of these two strata. The vehicle for the vitamins and the placebo, was a lipid-based paste provided in jars pre-labeled by the producer with a subject identification number and no indication on study group. A scientist at the University of Bergen, who was otherwise not involved in the study, provided the randomization list using Stata Version 10 (StataCorp, College Station, TX, USA) linking the unique child identification number with the intervention group. The subject identification number was the only indication that could link the paste to the study group. Ensuring double blinding, the placebo and the vitamin supplements were identical in appearance and taste and the allocation was masked to the participants as well as the study team throughout the data collection period.

Inclusion and exclusion criteria, as well as the other study procedures were the same for the main study and the current sub study.

### Interventions

We have previously demonstrated that gastrointestinal illnesses as well as folate and vitamin B12 deficiency is common in this population [4, 16, 17]. We therefore decided to provide folic acid and vitamin B12 at doses that were approximately twice the recommended daily allowances. The lipid based paste was chosen because of its acceptability in similar populations and because it is a feasible way to provide vitamins and minerals to child populations without degradation or contamination. The paste was prepared by NUTRISET, Ltd (Malaunay France). The interventions were given to the enrolled children daily by field workers. On Sundays and on public holidays the caregivers administered the supplementation to the children according to instructions. When families were travelling, field workers provided the supplement for the planned travel period in smaller units. All children across study groups were supplemented with one spoon (5 g) if they were 6 to 11 months, and two spoons (10 g) if they were 12 months and above. Each 10 g of supplement contained 54.1 kcal total energy, 0.7 g proteins and 3.3 g fat. For the intervention groups folic acid only, vitamin B12 only or vitamin B12/folic acid, the 10 g supplement also contained 150 μg folic acid or 1.8 μg vitamin B12 (as cyanocobalamin), or the combination of both. For the younger children, the 5 g supplement contained half of the vitamin doses of the older children. All children received the intervention for six months.

### Developmental assessment

Development was assessed at the end of the study following six months supplementation. The timing of the assessments was identical for all children in the sub study. Development was assessed using the ASQ-3, a developmental screening tool constructed in the US [18]. The ASQ-2^nd^ ed. has been validated against a developmental assessment tool in North India, and found to have good test characteristics for detecting developmental delay in this setting [19]. The ASQ-2 has also been used as an outcome measure in epidemiological studies worldwide [20–22], where both continuous and dichotomous outcomes (cut-offs) have been reported.

The ASQ-3 consists of age-appropriate questionnaires, all containing 30 items divided into five subscales: Communication, Gross motor, Fine motor, Problem-solving and Personal-social, summing up to five subscale scores (range 0 to 60) and a total score (range 0 to 300). Eleven forms (for age 12–36 months) were translated to Hindi following official recommendations [23], and items not suited for the cultural setting were identified and slightly adjusted [24]. In the translated ASQ-3 version, the standardized alphas for the total ASQ-3 scores were strong, indicating an overall acceptable internal consistency [24].

Three field supervisors were trained to administer the ASQ-3 directly with the child at the research clinic in presence of caregivers [25]. The examiners elicited the relevant skills from the child during sessions using standardized materials. The caregiver served as an important contributor in supporting the child, eliciting behaviors and gave relevant information of the child’s development when necessary. The three field supervisors were trained by the first author, a clinical child psychologist with experience in training and the assessment of infants and young children. During the 11 days of training, the field supervisors were standardized in performing the procedure, and they reached a high inter-observer agreement both during training and in a separate quality control where 10% of the observations were done in duplicate throughout the study [24].

To assess the caregiver`s promotion of child development we carefully selected two questions from the standardized assessment tool Home Observation for Measurement of the Environment (HOME) [26] that were asked the caregivers during the session. One question was on “Mother`s belief that child`s behavior can be modified” and one was on “Mother`s encouragement of developmental advances”.

### Growth and biochemical markers

Trained field supervisors measured weight and length at baseline and after six months of supplementation. Weight was measured to the nearest 50 g using Digitron scales. Length was measured using locally manufactured infantometers reading to the nearest 0.1 cm.

Venous blood samples were obtained at baseline for all children, and at end study in a subsample of randomly selected blocks (94 children for the subsample). Three mL of blood was collected into an evacuated tube containing EDTA (BD, Franklin Lakes, NJ, USA). Immediately following blood sampling, plasma was separated from the blood cells by centrifugation at room temperature (450 x g x 10 min), transferred into storage vials and stored at -20 0C until analysis. Plasma tHcy was analyzed using commercial kits (Abbott Park, IL, USA) [27]. Plasma concentrations of vitamin B12 and folate were determined by microbiological assays using a chloramphenicol-resistant strain of *Lactobacillus casei* and colistin sulfate-resistant strain of *Lactobacillus leichmannii*, respectively [28].

### Power calculations

We included the last 422 enrollments in this sub-study. The power to detect a standardized mean difference (based on a t-test) of 0.4 (i.e. 20 points in total ASQ-3 scores) and 0.5 (25 points in total ASQ-3 scores) between the placebo and any of the treatment groups was 83 and 95 per cent, respectively. In these calculations, which were done by the “power” command in Stata, we used a two-sided alpha error of 0.05.

### Statistics and data management

The data was entered twice by two data entry clerks followed by validiation by a computer manager. A total of 0.21% of the ASQ-3 responses were missing. For missing items an adjusted total score was computed by dividing the total subscale score by the number of completed items in the scale [29]. This number was then added depending on the amount of items missing. Height-for-age, weight-for-age and weight-for-height z scores were calculated using the most recent WHO growth charts [30]. The ASQ-3 scores for the total sample are presented as means (SD). We used linear regression to compare the intervention groups: vitamin B12, folic acid and vitamin B12/folic acid against the placebo on a continuous scale. We also used multiple logistic regression on the total and subscale ASQ-3 scores categorized on the 25th percentile. In these models, we also examined the effects in various predefined subgroups based on the following baseline characteristics (cut-offs in brackets): age (<12 months), stunting (< -2 z scores height/length-for-age), wasting (< -2 z scores weight-for-height/length), being underweight (< -2 z scores weight-for-age), low plasma vitamin B12 (<200 pmol/L), low plasma folate (<7.5 nmol/L), and high plasma tHcy (>10 μmol/L). This is the same approach for presenting main and subgroup effects as we used when presenting the effect of the interventions on the incidence of infections [15]. For the subgroup models, we adjusted for sex, age, breastfeeding status, height-for-age z-scores and mother´s encouragement of developmental advances. Post hoc, we examined the effect in an additional subgroup: ≤ 24 months vs. > 24 months at end study (corresponding to ≤18 months vs. >18 months at enrollment). We also performed the overall analyses adjusting for important baseline factors such as sex, gender, breastfeeding status, height-for-age, weight-for-age z-scores and log transformed family income. We included interaction terms in the models to measure whether the effects between the subgroups were significantly different. In these models, we also measured the interaction between folic acid and vitamin B12 supplementation. We calculated standardized mean differences by dividing the mean differences by the overall SDs of the different outcomes. Statistical analyses were performed in Stata, version 13 (Stata corporation, College Station, TX). All analyses were done following an intention-to-treat protocol. *P*< 0.05 (two-tailed) was considered significant.

## Results

**Fig 1:**
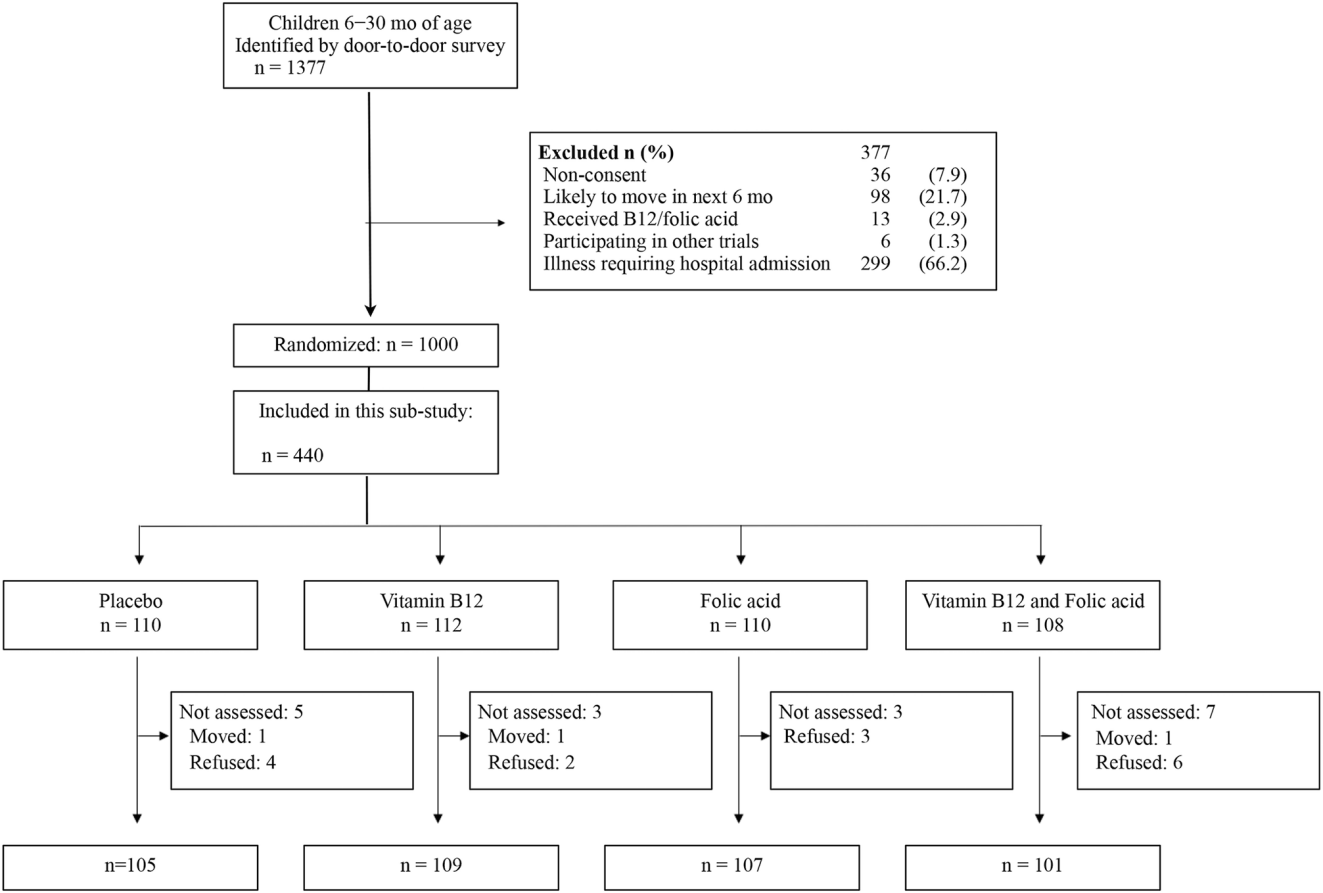
Trial profile of a randomized, placebo controlled trial on the effect of vitamin B12 and/or folic acid administration on development in 6–30 months old North Indian children.

**Fig 1** shows the flow of the participants through the study. Among the 1000 children randomized into the main study, the last 440 enrollments were included for developmental assessment. Three children were not available for assessment and 15 did not wish to participate, hence the final number of participants was 422. Baseline characteristics for the children in the four intervention groups are presented in **Table 1**. As reported from the main study, adherence was excellent and 96% of the scheduled doses were ingested [15]. Furthermore, compared to the placebo group, plasma vitamin B12 concentrations increased substantially in the group of children that received vitamin B12 alone or in combination with folic acid. Likewise, plasma folate levels increased significantly in children who received folic acid alone or in combination with vitamin B12 (**Table 2**).

**Table 1:** Baseline characteristics of the 422 children age 6–30 months.

	Placebo (n = 105)	Vitamin B12 (n = 109)	Folic Acid (n = 107)	Vitamin B12 & Folic Acid (n = 101)
**Child characteristics**				
Age, months	16.2 ± 7.4^1^	15.3 ± 6.6	15.8 ± 7.6	15.2 ± 6.6
6 − 11 months, n (%)	36 (36.2)	34 (31.2)	35 (32.7)	34 (33.7)
12 − 30 months, n (%)	69 (63.8)	75 (68.8)	72 (67.3)	67 (66.3)
Boys, n (%)	62 (59.0)	52 (47.7)	51 (47.7)	51(50.5)
Still breastfed, n (%)	92 (87.6)	97 (89.0)	88 (82.2)	87 (87.0)^2^
Z scores:				
Weight-for-height (WHZ)	-1.0 ± 0.9	-0.8 ± 0.9	-0.7 ± 1.0	-0.8 ± 0.9
Height-for-age (HAZ)	-1.9 ± 1.2	-1.7 ± 1.2	-1.7 ± 1.2	-1.7 ± 1.0
Weight-for-age (WAZ)	-1.7 ± 1.0	-1.5 ± 1.0	-1.4 ± 1.1	-1.4 ± 0.9
Wasted (<-2 WHZ), n (%)	15 (14.3)	12 (11.0)	8 (7.5)	7 (6.9)
Stunted (<-2 HAZ), n (%)	47 (44.8)	45 (41.3)	43 (40.2)	34 (33.6)
Underweight (<-2 WAZ), n (%)	42 (40.0)	28 (25.7)	36 (33.6)	25 (24.8)
**Family characteristics**				
Annual family income (INRx1000)^3^	72 (48, 120)	82 (60, 120)	72 (60, 126)	84 (62, 140)
Has television, scooter or cooler, n (%)	90 (85.7)	98 (89.9)	98 (91.6)	91 (90.1)
Living in joint family, n (%)	45 (42.9)	44 (40.4)	54 (50.5)	51 (50.5)
Family size	5.8 ± 2.4	5.8 ± 2.9	5.8 ± 2.4	5.9 ± 2.4
Age of mother, years	25.8 ± 4.6	26.5 ± 8.2	25.0 ± 3.6	25.3 ± 3.9
Mothers years of schooling n (%)				
No schooling (>5)	22 (21)	30 (27.5)	24 (22.4)	22 (21.8)
Primary (5 years complete)	65 (61.9)	50 (45.9)	46 (43)	48 (47.5)
Middle (10 years complete)	6 (5.7)	13 (11.9)	9 (8.4)	11 (10.9)
Higher (>10 years)	12 (11.4)	16 (14.7)	28 (26.2)	20 (19.8)
Fathers years of schooling				
No schooling (>5)	16 (15.2)	13 (11.9)	13 (12.2)	11 (10.9)
Primary (5 years complete)	36 (34.3)	36 (33.1)	41 (38.3)	39 (38.6)
Middle (10 years complete)	26 (24.8)	37 (33.9)	23 (21.5)	27 (26.7)
Higher (>10 years)	27 (25.7)	23 (21.1)	30 (28)	24 (23.8)
Mothers who work, n (%)	6 (5.7)	9 (8.3)	8 (7.5)	3 (2.7)
Attending Anganwadi centre^4^, n (%)	6 (5.8)	13 (11.9)	12 (11.2)	9 (8.9)
**Folate and vitamin B12 status at baseline**				
Vitamin B12 < 200 pmol/L, n (%)	36 (34.3)	36 (33.0)	39 (36.5)	27 (26.7)
Folate < 7.5 nmol/L, n (%)	27 (25.7)	38 (34.8)	30 (28.0)	36 (35.6)
tHcy > 10 μmol/L, n (%)	67 (63.5)	60 (53.21)	62 (57.9)	58 (57.4)

^1^ Mean±SD all such values.

^2^ Missing information from 1 child.

^3^ Indian Rupees in median (Interquartile range).

^4^Childcare centre.

**Table 2:** Concentrations of markers of vitamin B12 and folate status and change compared to the placebo.

	Placebo *(n = 105)*		B12 *(n = 109)*		Folic acid *(n = 107)*		B12 & Folic acid *(n = 101)*	
**Baseline**	**Median**	**IQR** ^**1**^	**Median**	**IQR**	**Median**	**IQR**	**Median**	**IQR**
Plasma vitamin B12 (pmol/L)	260	(170–353)	269	(178–419)	286	(180–473)	307.0	(198–439)
Plasma folate (nmol/L)	10.4	(7.0–18.4)	9.1	(6.1–18.2)	11.8	(6.7–20.3)	9.7	(6.1–17.8)
Plasma tHcy (μmol/L)	12.7	(9.1–17.2)	10.6	(8.7–15.0)	11.8	(8.7–15.2)	10.4	(8.2–13.5)
**End of study**	*(n = 24)*		*(n = 22)*		*(n = 24)*		*(n = 24)*	
Plasma vitamin B12 (pmol/L)	285	(199–368)	362	(271–708)	297	(225–405)	446	(302–565)
Plasma folate (nmol/L)	16.6	(11.9–22.5)	11.8	(8.0–19.3)	49.0	(28.6–64.0)	50.5	(31.2–78.4)
Plasma tHcy (μmol/L)	12.4	(10.3–15.5)	8.0	(6.5–9.5)	8.1	(6.7–11.7)	7.6	(5.8–10.0)
**Change from baseline to end of study compared to placebo**			**Mean diff.** ^**2**^	**95% CI** ^**3**^	**Mean diff.**	**95% CI**	**Mean diff.**	**95% CI**
Vitamin B12 (pnmol/L)	0		118	(17, 219)	6	(-93, 105)	102	(4, 202)
Folate (nmol/L)	0		-3.2	(-15.0, 8.6)	28.5	(17.0, 40.0)	34.3	(22.8, 45.7)
tHcy (μmol/L)	0		-1.7	(-5.0, 1.5)	-1.2	(-4.5, 2.1)	-5.3	(-8.6, -2.1)

^1^ Interquartile range.

^2^ Mean difference change in concentration from baseline.

^3^ 95% Confidence interval.

### The effect of the intervention on development

Overall, the total ASQ-3 score was 12.6 (95% CI -1.1, 26.3) (*P* = 0.071) points higher in the group of children who received six months of vitamin B12/folic acid supplementation compared to those who received placebo (**Table 3**). Higher scores in the vitamin B12/folic acid supplementation group were also observed for the Gross motor subscale (*P* = 0.001) and the Problem-solving subscale (*P* = 0.048), while no significant effect was observed for the Communication, Fine motor and Personal social subscales **(Table 3).** The mean standardized effect sizes for the Total, Gross Motor and the Problem-solving subscales were 0.25 (95% CI -0.02, 0.53), 0.46 (95% CI 0.19, 0.73) and 0.28 (95% CI 0.02, 0.54) SD units respectively.

**Table 3:** The effect of vitamin B12 and/or Folic acid on ASQ-3 total and subscale scores.

	Placebo *(n = 105)*	B12 *(n = 109)*			Folic acid *(n = 107)*			B12 & Folic acid *(n = 101)*		
	Mean (SD)	Mean (SD)	Mean Diff.^1^	95%CI^2^	Mean (SD)	Mean Diff.	95%CI	Mean (SD)	Mean Diff.	95%CI
**Total ASQ-3**	228.0 ± 47.2	230.6 ± 52.2	2.6	(-10.8, 16.0)	228.6 ± 55.8	0.6	(-12.7, 14.3)	240.6 ± 43.2	12.6	(-1.1, 26.3)
**Subscale**										
Communication	47.4 ± 14.8	47.8 ± 15.9	0.4	(-2.8, 4.6)	48.2 ± 15.7	0.8	(-3.4, 5.0)	47.9 ± 15.4	0.5	(-3.8, 4.7)
Gross motor	42.8 ± 15.2	46.8± 13.6	4.0	(0.3, 7.8)*	45.8 ± 15.3	3.0	(-0.8, 6.8)	49.3 ± 11.4	6.5	(2.7, 10.3)**
Fine motor	47.6 ± 13.2	47.1 ± 13.3	-0.5	(-4.1, 3.1)	44.5 ± 14–9	-3.1	(-6.8, 0.5)	47.6 ± 12.2	0	(-3.7, 3.7)
Problem-solving	44.3 ± 14.1	43.1 ± 15.6	-1.2	(-4.9, 2.5)	43.9 ± 13.3	-0.4	(-4.1, 3.3)	48.1 ± 11.5	3.8	(0.0, 7.6)*
Personal social	45.9 ± 12.4	45.7 ± 12.5	-0.2	(-3.5, 3.2)	46.4 ± 12.9	0.5	(-2.8, 3.9)	47.7 ± 12.0	-1.8	(-1.6, 5.2)

*p<0.05.

**p<0.01.

^1^ Mean difference in total ASQ scores from Placebo.

^2^ 95% Confidence interval.

In groups that either received folic acid or vitamin B12 alone, we did not find significant differences from placebo with the exception of children who received vitamin B12 without folic acid: The gross motor scale was 4.0 (95% CI 0.3, 7.8) points higher in the vitamin B12 group than in the placebo group **(Table 3)**. The corresponding effect size was 0.29 (95% CI 0.02, 0.55) SD units.

We repeated the analyses using logistic regression after dichotomizing the outcomes at the 25 percentiles. Similar results were observed as for the linear regression reported above **(Table 4)**. We also undertook these comparisons adjusting for baseline differences; the adjusted analyses resulted in only modest changes to the estimates and the levels of significance (**S1 and S2 Tables**).

**Table 4:** ORs^1^ (95% CIs^2^) for being in the lower quartile of ASQ-3 total and subscale scores compared with placebo.

	Placebo (n = 105)	B12 (n = 109)		Folic acid (n = 107)		B12 & Folic acid (n = 101)	
	OR	OR	95% CI	OR	95% CI	OR	95% CI
**Total ASQ-3**	1	0.66	(0.37, 1.19)	0.75	(0.42, 1.34)	0.53	(0.29, 0.99)*
**Subscale**							
Communication	1	0.95	(0.53, 1.68)	0.89	(0.50, 1.59)	0.88	(0.49, 1.59)
Gross motor	1	0.74	(0.43, 1.28)	0.74	(0.43, 1.28)	0.39	(0.22, 0.71)**
Fine motor	1	0.99	(0.56, 1.74)	1.57	(0.90, 2.73)	0.93	(0.52, 1.66)
Problem-solving	1	0.97	(0.57, 1.67)	0.83	(0.48, 1.43)	0.52	(0.29, 0.93)*
Personal social	1	0.98	(0.56, 1.71)	1.05	(0.60, 1.83)	0.72	(0.40, 1.28)

*p<0.05.

**p<0.01.

^1^ Odds Ratio.

^2^ 95% Confidence interval.

### Subgroup analyses

In the subgroup analyses we present the results from the logistic regression analyses. Stunted children that received six months of vitamin B12/folic acid supplementation had substantially and significantly reduced odds [Odds ratio (OR): 0.26 (95% CI 0.09, 0.78) (*P* = 0.016)] of being in the lowest quartile of the ASQ-3 score. This was also the case for children with elevated levels of tHcy at baseline with significantly reduced odds [OR: 0.38 (95% CI 0.16, 0.92) (*P* = 0.032)] of being in the lowest quartile. Based on the assumption that the period up to 24 months is a critical time for brain development, we also measured the effect of the interventions separately for children who were ≤24 months at end study vs. the older children. In children ≤24 months at the end of the study (≤ 18 months at enrollment), vitamin B12/folic acid supplementation led to significantly lower odds of being in the lower quartile of the ASQ-3 score (OR: 0.37, 95% CI 0.17–0.83, P = 0.015). These effects were not seen in children aged 19 to 30 months at enrollment (**Table 5**). None of the subgrouping variables significantly modified the effect of the interventions on the ASQ-3 scores, and the interaction between folic acid and vitamin B12 supplementation was not significant.

**Table 5:** ORs^1^ (95% CIs)^2^ for being in the lower quartile of ASQ-3 total in the intervention group compared with placebo in subgroups.^3^

		Placebo	Vitamin B12		Folic acid		B12 & Folic acid	
	N	OR	OR	95% CI	OR	95% CI	OR	95% CI
**Subgroups**								
** Age in months at baseline**								
6 to11 months	138	1	0.51	(0.17, 1.50)	0.79	(0.26, 2.39)	0.48	(0.16, 1.48)
12 to 30 months	283	1	0.74	(0.33, 1.63)	1.12	(0.50, 2.48)	0.59	(0.25, 1.36)
6 to 18 months	149	1	0.46	(0.22–0.99)*	0.73	(0.34–1.57)	0.37	(0.17–0.83)*
19 to 30 months	273	1	1.65	(0.49–5.60)	1.87	(0.55–6.33)	1.20	(0.33–4.43)
** Growth at baseline**								
Wasted	42	1	0.57	(0.06, 5.25)	4.29	(0.29, 62.61)	1.34	(0.11, 16.38)
Not wasted	379	1	0.67	(0.34, 1.32)	0.92	(0.47, 1.78)	0.53	(0.26, 1.08)
Stunted	169	1	0.48	(0.19, 1.22)	0.77	(0.29, 2.02)	0.26	(0.09, 0.78)*
Not stunted	252	1	0.95	(0.39, 2.34)	1.25	(0.51, 3.08)	1.11	(0.46, 2.71)
Underweight	131	1	0.54	(0.17, 1.64)	0.84	(0.29, 2.45)	0.64	(0.21, 1.97)
Not underweight	290	1	0.78	(0.36, 1.72)	1.05	(0.47, 2.35)	0.49	(0.21, 1.18)
** Biochemical markers at baseline**								
Vitamin B12 <200 pmol/L	137	1	0.48	(0.17, 1.38)	0.68	(0.24, 1.90)	0.61	(0.20, 1.85)
Vitamin B12 ≥200 pmol/L	284	1	0.87	(0.39, 1.95)	1.15	(0.51, 2.65)	0.59	(0.25, 1.38)
Folate <7.5 nmol/L	131	1	0.95	(0.28, 3.28)	1.44	(0.41, 5.13)	0.58	(0.16, 2.11)
Folate ≥7.5 nmol/L	290	1	0.65	(0.30, 1.38)	0.84	(0.40, 1.76)	0.60	(0.27, 1.32)
tHcy >10 μmol/L	247	1	0.66	(0.30, 1.46)	0.73	(0.33, 1.61)	0.38	(0.16, 0.92)*
tHcy ≤10 μmol/L	174	1	1.10	(0.37, 3.29)	1.86	(0.61, 5.67)	1.10	(0.37–3.27)

*p<0.05.

^1^Odds Ratio.

^2^95% Confidence interval.

^3^Adjusted for sex, age, breastfeeding status, stunting and Mother`s encouragement of developmental advances (dichotomus).

## Discussion

In our study, we found a borderline significant positive effect of receiving vitamin B12 and folic acid for six months on the total ASQ-3 scores. We also found a significant and positive effect of vitamin B12 and folic acid in the developmental domains of gross motor functioning and problem-solving skills. The effects of supplementation were most evident in children who were stunted, and in children who had high plasma tHcy consistent with poor folate and/or vitamin B12 status, and in children who completed the study before their 2^nd^ birthday [31]. Except for vitamin B12 in relation to gross motor functioning, there was no significant benefit of giving vitamin B12 or folic acid alone on early child development.

North Indians, including children, often have poor vitamin B12 status due to dietary practices (vegetarianism) and poverty [2]. Poor vitamin B12 status was also seen in the present study. In addition, a large proportion had low plasma folate, and nearly 60% of these young children had elevated tHcy at baseline. Children who received supplementation of vitamin B12 and folic acid for six months had substantial improvement in vitamin status and a reduction in plasma tHcy concentrations [15].

Through our assessment across developmental domains, we found that children who received supplementation of vitamin B12 and folic acid for six months had improved gross motor outcomes and problem-solving skills, while there were no significant changes in the domains of communication, fine motor and personal-social skills. The results are in line with previous observational studies that have documented an association between vitamin B12 status and aspects of development [9, 32]. However, the specific developmental domains that are related to vitamin B12 differ between studies and are not directly comparable due to methodological differences. For example, in the study in North Indian children, marginal vitamin B12 status was related to lower scores on the mental development index of the Bayley 2nd ed, but not to the scores on the psychomotor scale [9]. These scales are not directly comparable to the ASQ domains. The association between gross motor functioning and vitamin B12 has been documented across several studies, i.e. in a Dutch vegan study group, where vitamin B12 deficient infants suffered from slower gross motor and language development compared to non-deficient infants [32]. Further, in a recently published RCT, Norwegian infants with poor vitamin B12 status and who were referred to a pediatrician due to feeding problems had substantially and significantly improved feeding and gross motor functioning following a vitamin B12 injection [11]. These findings are consistent with the findings in the present study. However, in the study of Norwegian children, they did not assess other developmental domains. Furthermore, while the Norwegian study relates to the rare situation of a child being submitted to hospital for feeding problems, our data relate to associations of the general Indian population of people belonging to middle to poor socioeconomic classes.

Given the nature of the developing brain with periods of regional brain growth spurts and rapid maturation, it is challenging to compare results on specific developmental areas across age groups. Functions under development are particularly sensitive to influences, which may give rise to developmentally dependent outcomes [33, 34]. Consequently, we need to consider the timing of the exposure as well as the period of assessment [34, 35]. For instance, in the Dutch study of vegan families, findings indicate long-term consequences of early vitamin B12 deficiency on cognitive performance in adolescence [8]. In early childhood, these adolescents had significantly lower functioning in the areas of gross motor and language development compared to a control group [32]. In our study, we measured the short-term effects following six months supplementation. The time required for improvements to be detectable in the developing brain and possible to assess, may be domain specific [36]. Thus, although we did not find significant effects on communication skills, fine motor functioning and personal social abilities, an impact on these domains may require longer exposure time, or improvements may become apparent later in life. The interruption of myelination has been suggested as a possible mechanism linking vitamin B12 deficiency and adverse neurodevelopment [7]. Myelination facilitates communication in the brain and interruptions may influence the speed of conductance and thus the process of learning and acquisition of skills. Our result may indicate that the mechanisms of improved myelination give a more rapid effect in gross motor and problem-solving skills than for the other domains assessed. On the other hand, it has been argued that myelin repair is a slow process, and the improvements observed after short-term vitamin B12 supplement may be related to other effects such as increased energy-production in the central nervous system [37]. Thus, the improvements in the current study may be due to increased energy and attention, which are important factors for the performance both for gross motor abilities and problem-solving skills. Further research is required to achieve a deeper understanding of the specific consequences of vitamin B12 and folate deficiency on the developing brain.

Being a time of rapid growth, the period from gestation to 24 months of age is a sensitive period for brain development in which the brain is particularly susceptible to various exposures [33, 35, 38]. In support of this, there was a significant benefit of the vitamin B12 and folic acid supplementation in children who were ≤ 18 months at the start of supplementation and received all supplementation before they reach 24 months of age. Thus, our findings support that the timing of nutritional influence is of significance for the outcome of neurodevelopment.

The effect of vitamin B12 and folic acid supplementation on total ASQ-3 scores was most apparent in stunted children and in children with elevated tHcy. Childhood stunting is a proxy for several factors that are associated with poor neurodevelopment [39], consequently there might be a large potential for improvements among children who are stunted. The hypothesis of “functional isolation” where a malnourished child fails to elicit appropriate care and stimulation from the caregiver due to behavior symptoms such as irritability and apathy, may, in part, explain the effect in this subgroup [40]. Stunting could also reflect poor long-term vitamin B12 or folate intake, and the improved scores in children receiving vitamin B12 and folic acid may suggest that developmental delay in stunted children partly is related to low folate or vitamin B12 status. This possibility is supported by the finding that children with elevated levels of tHcy at baseline had a significant beneficial effect of vitamin B12 and folic acid supplementation on the total ASQ-3 score, while those with normal tHcy concentration did not. Stunting or elevated tHcy did not significantly modify the effect of the intervention on the total ASQ-3 score, but it should be noted that the trial was not powered to measure such interactions.

Our results give support to the hypothesis of the importance of vitamin B12 and folate for neurodevelopment and subsequent behavioral outcomes. Similarly, a recent report from the current study provides evidence for the importance of vitamin B12 for growth showing that poor vitamin B12 status in the children contributes to poor growth [41]. Clearly, there are several modifiable risk factors associated with adverse neurodevelopment other than vitamin B12 and folate deficiency [14]. There is sound evidence that adequate stimulation and responsive caregiving is crucial for healthy neurodevelopment as recently illustrated in a report from the current study [42]. Furthermore, biological risk factors such as childhood infections and chronic undernutrition may lead to adverse neurodevelopment and unfulfilled developmental potential [14]. Further research is needed to understand the role of vitamin B12 and folate as one important factor for neurodevelopment in the complex interrelation between brain development and environmental influences.

### Strength and weaknesses

To our knowledge, this study is the first RCT that has measured the effects of vitamin B12 and/or folic acid on child development. This is a well-conducted RCT with few losses to follow-up and excellent adherence to the supplementation [15]. Despite an optimal randomization procedure involving more than 400 children, there were some baseline differences in family characteristics and physical growth between the groups. These represent a limitation to the study that may confound our effect estimates. We have repeated the analyses adjusting for these and other relevant baseline characteristics such as sex, age, breastfeeding status, height-for age and weight-for-height z-scores and log transformed family income. These adjustments resulted in minor alterations in the effect estimates and, hence, the level of significance. However, the main finding of a beneficial effect of vitamin B12/folic acid on gross motor and problem-solving skills remained significant (**S1 and S2 Tables**). If we had measured development at baseline in addition to at end study, we could have estimated the change in ASQ scores throughout the supplementation period and thereby adjusted for baseline differences in the ASQ scores. This was not possible due to the timing of the parent project and the time it took to prepare the instruments for the current sub-study. Despite the fact that each study group consisted of more than 100 children and that we carefully adjusted for baseline differences and other relevant variables, we cannot rule out residual confounding and our findings need to be confirmed before treatment recommendations can be made.

The ASQ-3 is a screening tool for the assessment of developmental delay constructed in the US with binary cut-offs, but has been used to measure developmental status on a continuous scale in several studies, as in the present study. It should be noted, that the questionnaires have not yet been validated for this purpose. We translated 11 ASQ-3 forms to Hindi particularly for this study, and the translated and adjusted ASQ-3 served as an easily administered and cost efficient assessment tool [24]. However, alpha values indicated questionable internal consistency in a few subscales and age categories. Poor internal consistency can be due to constant items (lack of variability of the responses) or random errors. In a randomized trial, random errors should be similar in the study groups and accordingly not result in biased effect estimates. More comprehensive assessment tools, such as the Bayley scales, or tools for social emotional functioning, could have added a broader picture of the children`s skills and abilities. Finally, more advanced neuroimaging techniques might have identified unique changes to the developing brain in early childhood.

## Conclusion

In a RCT of supplementation with vitamin B12 and folic acid in six to 30 months old children we found beneficial effects on neurodevelopment as assessed by a screening tool in the domains of gross motor functioning and problem solving skills. These results need to be confirmed in other populations with more comprehensive assessment tools, and further research is recommended on the long-term effects of marginal vitamin B12 and folate status in the developing brain.

## Supporting Information

S1 CONSORT Checklist (PDF)

S1 Table The effect of vitamin B12 and/or Folic acid on ASQ-3 total and subscale scores, adjusted for baseline characteristics. (DOCX)

S2 Table ORs (95% CIs) for being in the lower quartile of ASQ-3 total and subscale scores compared with placebo, adjusted for baseline characteristics. (DOCX)

S1 Protocol Study Protocol. (PDF)

S2 Protocol ClinicalTrials.Gov Protocol registration receipt. (PDF)
