# Supplementary material for: Vitamin B12 and Folic Acid Improve Gross Motor and Problem-Solving Skills in Young North Indian Children: A Randomized Placebo-Controlled Trial
Source: PLoS One. 2015 Jun 22;10(6):e0129915. doi: 10.1371/journal.pone.0129915 (PMC4476750; doi:10.1371/journal.pone.0129915)
Supplement: S1 Table — (DOCX) [file pone.0129915.s002.docx]

**Table S1. The effect of vitamin B12 and/or Folic acid on total ASQ and subscale scores adjusting for baseline characteristics^1^**

|  | |  | **Placebo**  ***(n = 105)*** |  | **B12**  ***(n = 109)*** | | |  | **Folic acid**  ***(n = 107)*** | | |  | **B12 & Folic acid**  ***(n = 101)*** | | |
| --- | --- | --- | --- | --- | --- | --- | --- | --- | --- | --- | --- | --- | --- | --- | --- |
|  | |  | **Mean (SD)** |  | **Mean (SD)** | **Mean**  **Diff.**^2^ | **95%CI^3^** |  | **Mean (SD)** | **Mean**  **Diff.** | **95%CI** |  | **Mean (SD)** | **Mean**  **Diff.** | **95%CI** |
| **Total ASQ-3** | | | 228.0 ± 47.2 |  | 230.6 ± 52.2 | -0.2 | (-13.1, 12.6) |  | 228.6 ± 55.8 | -4.7 | (-17.7, 8.4) |  | 240.6 ± 43.2 | 9.4 | (-3.7, 22.6) |
| **Subscale** | | |  |  |  |  |  |  |  |  |  |  |  |  |  |
|  | Communication | | 47.4 ± 14.8 |  | 47.8 ± 15.9 | _-0.3 | (-4.4, 3.6) |  | 48.2 ± 15.7 | -0.9 | (-4.9, 3.2) |  | 47.9 ± 15.4 | -0.5 | (-4.6, 3.5) |
|  | Gross motor | | 42.8 ± 15.2 |  | 46.8± 13.6 | 3.4 | (-0.2, 7.0) |  | 45.8 ± 15.3 | 1.7 | (-2.0, 5.4) |  | 49.3 ± 11.4 | 5.5 | (1.8, 9.5)** |
|  | Fine motor | | 47.6 ± 13.2 |  | 47.1 ± 13.3 | -0.8 | (-4.4, 2.8) |  | 44.5 ± 14-9 | -3.7 | (-7.4, -0.1) |  | 47.6 ± 12.2 | -0.3 | (-4, 3.4) |
|  | Problem-solving | | 44.3 ± 14.1 |  | 43.1 ± 15.6 | -1.6 | (-5.1, 2.0) |  | 43.9 ± 13.3 | -1.5 | (-5.1, 2.2) |  | 48.1 ± 11.5 | 3.4 | (-0.3, 7.1) |
|  | Personal social | | 45.9 ± 12.4 |  | 45.7 ± 12.5 | -0.9 | (-4.1, 2.4) |  | 46.4 ± 12.9 | -0.3 | (-3.6, 3.0) |  | 47.7 ± 12.0 | 1.3 | (-2.1, 4.6) |

*p<0.05, **p<0.01

^1^ Adjusted for sex, age, breastfeeding status, height-for-age and-weight for-age z scores and log transformed family income

^2^ Mean difference change in total ASQ-scores from Placebo

^3^ 95% Confidence interval
