# Supplementary material for: Vitamin B12 and Folic Acid Improve Gross Motor and Problem-Solving Skills in Young North Indian Children: A Randomized Placebo-Controlled Trial
Source: PLoS One. 2015 Jun 22;10(6):e0129915. doi: 10.1371/journal.pone.0129915 (PMC4476750; doi:10.1371/journal.pone.0129915)
Supplement: S2 Table — (DOCX) [file pone.0129915.s003.docx]

**Table S2. ORs^2^ (95% CIs^3^) for being in the lower quartile of ASQ-3 total and subscale scores compared with placebo adjusting for baseline characteristics**^3^_._

|  | | **Placebo (n=105)** | **B12 (n=109)** | | **Folic acid (n=107)** | | **B12 & folic acid (n=101)** | |
| --- | --- | --- | --- | --- | --- | --- | --- | --- |
|  | | **OR** | **OR** | **95% CI** | **OR** | **95% CI** | **OR** | **95% CI** |
| **Total ASQ-3** | | 1 | 0.71 | (0.38, 1.33) | 0.93 | (0.50, 1.74) | 0.58 | (0.30, 1.12) |
| **Subscale** | |  |  |  |  |  |  |  |
|  | Communication | 1 | 1.03 | (0.56, 1.89) | 1.09 | (0.59, 2.01) | 1.00 | (0.54, 1.87) |
|  | Gross motor | 1 | 0.80 | (0.45, 1.42) | 0.85 | (0.48, 1.51) | 0.44 | (0.24, 0.81)** |
|  | Fine motor | 1 | 1.03 | (0.58, 1.85) | 1.74 | (0.98, 3.10) | 0.95 | (0.52, 1.73) |
|  | Problem-solving | 1 | 1.00 | (0.58, 1.76) | 0.90 | (0.51, 1.60) | 0.52 | (0.29, 0.94)* |
|  | Personal social | 1 | 1.08 | (0.65, 2.05) | 1.15 | (0.65, 2.05) | 0.76 | (0.42, 1.38) |

*p<0.05, **p<0.01

^1^ Odds Ratio

^2^ 95% Confidence interval

^3^ Adjusted for sex, age, breastfeeding status, height-for-age and-weight for-age z scores and log transformed family income
