# Supplementary material for: Vitamin B12 and Folic Acid Improve Gross Motor and Problem-Solving Skills in Young North Indian Children: A Randomized Placebo-Controlled Trial
Source: PLoS One. 2015 Jun 22;10(6):e0129915. doi: 10.1371/journal.pone.0129915 (PMC4476750; doi:10.1371/journal.pone.0129915)
Supplement: S1 Protocol — (PDF) [file pone.0129915.s004.pdf]

Project Application—Thrasher Research Fund

Title (Provide a descriptive title rather than a general title)

Routine administration of folic acid and vitamin B12 to prevent childhood infections.

**Principal Investigator**		**Co Investigator(s)**
Specify one person who is responsible to the Thrasher Research Fund for the scientific and/or technical work of the project. This individual is responsible for grant-related correspondence and expenditures.		List additional individuals who are responsible for the scientific and/or technical work of the project.
Name and address<br>Tor A Strand, MD, PhD<br>Centre for International Health<br>University of Bergen<br>5021 Haukeland Hospital<br>Norway		Name(s) and institution(s)<br>Dr. Sunita Taneja (Co principal investigator)<br>Society for Essential Health Action and Training, New Delhi, India<br>Dr.Tivendra Kumar (Co investigator)<br>Society for Applied Studies , New Delhi, India<br>Professor. Nita Bhandari (Co investigator)<br>Society for Essential Health Action and Training, New Delhi, India<br>Professor Gagandeep Kang (Co investigator)<br>Christian Medical College<br>Vellore, India
Telephone<br>+ 47 55 97 49 80	Fax<br>+ 47 55 97 49 80	
E-mail<br>tor.strand@cih.uib.no		

Human Subjects? If yes, complete the enclosed Protection of Human Subjects form. Form Status

☒ Yes ☐ No ☐ Included ☐Pending ☐NA

Animals Used? If yes, document compliance with Animal Welfare Assurance laws. Form Status

☐ Yes ☒ No ☐ Included ☐Pending ☐NA

Project Period The entire project may not exceed 3 years. The project start date will be determined in consultation with Thrasher Research Fund staff.

Approximate start date (month and year)	Approximate end date (month and year)
January 2008	December 2010

Total Budget Request

$ 398,694

Performance Site(s) Indicate where the work described in the "Experimental Design and Methodology" section will be performed. Please provide specific organization names and their complete addresses, including zip/postal code, telephone number, fax number, and email.

Organization name and address	Organization name and address
Society for Essential Health Action and Training<br>B10 Soami Nagar, New Delhi-110016, India	Society for Applied Studies,<br>45, Kalu Sarai, New Delhi-110016, India<br>Christian Medical College<br>Vellore, India

Supervising Institution Name the *one* organization that will be legally and financially responsible and accountable for the use and disposition of any funds awarded on the basis of this application

Supervising institution name and address	
Centre for International Health<br>University of Bergen<br>5021 Haukeland Hospital, Bergen, Norway	Phone +47 55 97 49 80<br>Fax +47 55 97 49 79<br>Email cih@cih.no

Official Signatures for Supervising Institution

We, the undersigned, certify that the statements herein are true and complete to the best of our knowledge and that facilities are available for the proposed research. We will comply with the Thrasher Research Fund's Conditions of Grant and requirements for reporting that are in effect at the time of the award.

Name and title of principal investigator<br>Tor A Strand, postdoctoral researcher	Signature	Date (mm/dd/yy)<br>06/29/07
Name and title of department chairman, if applicable<br>Halvor Sommerfelt, Acting director, Centre for Int. Health	Signature	Date (mm/dd/yy)<br>06/29/07
Name and title of official from supervising institution<br>Kristen Haugland, Research Director, University of Bergen	Signature	Date (mm/dd/yy)<br>06/29/07

Protection of Human Subjects Assurance/Certification/Declaration
Thrasher Research Fund

Policy A research project involving human subjects sponsored by a U.S.-based institution that is not exempt from HHS regulations may not be funded unless an institutional review board (IRB) has reviewed and approved the activity in accordance with Section 474 of the Public Health Service Act as implemented by Title 45. Part 46, of the Code of Federal Regulations (45 CFR 46-as revised). The applicant institution must submit certification of IRB approval to the Thrasher Research Fund unless the applicant institution has designated a specific exemption under Section 46.101(b) that applies to the proposed research project. Institutions with an assurance of compliance on file with HHS that covers the proposed project should submit certification of IRB review and approval with each application. In the case of institutions that do not have an assurance of compliance on file with HHS covering the proposed project, certification of IRB review and approval must be submitted within 30 days of the receipt of a written request from HHS for certification. Documentation of IRB approval from all project-related participating institutions must be included.

Title of application Routine administration of folic acid and vitamin B12 to prevent childhood infections.

Principal investigator Tor A Strand, MD PhD

Investigational New Drug Exemption If more than one is involved, list others under notes below.

Food and Drug Administration required information In accordance with 45 CFR 46.121, if an application is made to HHS requiring certification and involving use of an investigational new drug or device, additional information is required. Thirty (30) days must elapse between date of receipt by FDA of Form FD-1571 and use of the drug, unless the 30-day delay period is waived by FDA (21 CFR 312.1 ©a[a] ©2[a]).

Sponsor name University of Bergen, Norway

Drug name N/A

**Date of end of 30-day expiration or waiver**	**Number issued**

HHS Assurance Status

	Assurance identification number	IRB identification number
☒ This institution has an approved assurance of compliance on file with HHS which covers this activity.	FWA00009490 and FWA00012761	IRB00001873 and IRB00006362

☒ No assurance of compliance which applies to this activity has been established with HHS, but upon request the applicant institution will provide written assurance of compliance and certification of IRB review and approval in accordance with 45 CFR 46.

Certification of IRB Review or Declaration of Exemption

☐ This activity has been reviewed and approved by an IRB in accordance with the requirements of 45 CFR 46, including its relevant subparts. This certification fulfills, when applicable, requirements for certifying FDA status for new investigational drug or device.

**Date of IRB approval** (If approval is pending, write "pending." Follow-up certification is required.) Pending	☒ Full Board Review ☐ Expedited Review
☐ This activity contains multiple projects, some of which have not been reviewed. The IRB has granted approval on condition that all projects covered by 45 CFR 46 will be reviewed and approved before they are initiated and that appropriate further certification (Form HHS 5156) will be submitted.	☐ Human subjects are involved, but this activity qualifies for exemption under 46.101(b) in accordance with paragraph______ [insert paragraph number of exemption in 46.101(b), 1 through 5], but the institution did not designate that exemption on the application.

Each official signing below certifies that the information provided on this form is correct and that each institution assumes responsibility for assuring required future reviews, approvals, and submissions of certification.

Application institution		Cooperating institution	
Name, address, telephone and e-mail		Name, address, telephone and e-mail	
Name and title of official		Name and title of official	
Signature of official	Date	Signature of official	Date

Notes
The sponsoring institution (University of Bergen) is not an U.S based institution .

Principal Investigator Tor A Strand		
Supervising institution University of Bergen	Duration (years) 3	Total budget request 398694
Project location New Delhi, India		
Project name: Routine administration of folic acid and vitamin B12 to prevent childhood infections.		

Lay Abstract (Do not exceed one page.)

The Lay Abstract should read in such a way that a person not familiar with the proposed research could understand the objectives and intended practical outcomes of the project. The abstract serves as a succinct and accurate description of the proposed work when separated from the application. This information is directed specifically to the Executive Committee members as they make final funding decisions. (Type below line)

Rationale Pneumonia and diarrhea are among the leading causes of poor health and death in young children of developing countries.

Many of these children have inadequate intakes of several vitamins and minerals. Folate and vitamin B12 are important for normal function of the immune system. Deficiencies of these vitamins are often part of general malnutrition and might be responsible for the excess morbidity and mortality seen in malnourished children. In a recent cohort study in almost 2,500 Indian children we demonstrated that those with poor folate status had higher rates of diarrhea and pneumonia. This study also showed that children that were not breastfed had poor folate status and our analyses suggested that the effect of breastfeeding in preventing respiratory and gastrointestinal infections could be explained by the folate content of breast milk. The finding that poor folate status is related to increased susceptibility to childhood infections needs to be verified in properly conducted clinical trials in populations where folate deficiency is prevalent.

Hypothesis: Supplementation of two recommended daily allowances (RDA) of folic acid with or without simultaneous administration of vitamin B12 reduces the rates of acute lower respiratory tract infections (ALRI), clinical pneumonia and diarrhea.

Design/Methods We will conduct a preventive randomized placebo controlled clinical trial of folic acid and vitamin B12 supplementation in 1000 children aged 6 to 30 months living in a low to middle-income socioeconomic setting in New Delhi, India. Eligible children will be identified through a house-to-house survey. We will include 4 to 5 children every day who will be randomized to 4 treatment groups. These children will be given: 2 RDA of both vitamin B12 and folic acid, 2 RDA of folic acid only, 2 RDA of vitamin B12 only, or placebo. The supplements will be given daily for 6 months.

Data We will compare how frequent ALRI and diarrhea occur in the four different groups. This approach with four groups makes it possible to examine if folic acid in children that are given B12 and in those that are not. The latter analysis is important, as utilization of folates requires appropriate vitamin B12 status. Furthermore, this design also enables us to measure the effect of daily vitamin B12 supplementation on childhood morbidity in a population with a high prevalence of vitamin B12 deficiency.

Potential benefits: If folic acid treatment reduces the risk of these common infections in early childhood, it could reduce the high infant death rate in developing countries. Furthermore, by giving folic acid, it will be possible to counteract some of the negative effects faced by children that cannot be breastfed. Since folic acid with or without vitamin B12 is cheap, safe and without serious side effects, this will be particularly useful in developing countries.

Principal Investigator Tor A Strand		
Supervising institution University of Bergen	Duration (years) 3	Total budget request 398694
Project location New Delhi, India		
Project name Routine administration of folic acid and vitamin B12 to prevent childhood infections.		

Scientific Abstract (Do not exceed one page.)

Summarize the proposal's objectives and specific aims, making reference to how the project relates to health, and describe concisely the methodology for achieving these goals. The abstract serves as a succinct and accurate description of the proposed work when separated from the application. This information is directed specifically to the Advisory Committee and peer reviewers who are conversant with the area of the proposed research.

Rationale The defence against infections relies on the ability of the immune cells to proliferate and differentiate and on effective renewal of the epithelial linings. Folate and vitamin B12 play a crucial role in DNA and protein synthesis, suggesting that processes in which cell proliferation is essential may be impaired by poor status. Indeed, macroscopic disruption of the epithelial linings occurs with anti-folate treatment and several facets of the immune system are affected by folate and vitamin B12 deficiency. The phagocytic and bacteriocidal ability of polymorphonuclear leukocytes is poor in individuals with severe folate deficiency and improves with folate replenishment. Furthermore, the thymus and cell-mediated immunity, the blastogenic response of T lymphocytes to certain mitogens and the antibody responses to several antigens is reduced in folate-deficient individuals. Thus, deficiencies of these nutrients have negative consequences for the resistance against infections.

Pneumonia and diarrhea are among the leading causes of morbidity and mortality in children below five years of age in developing countries. Many of these children have inadequate intakes of energy as well as vitamins and minerals. Deficiencies of folate and vitamin B12 are often part of general malnutrition and might in part be responsible for the excess morbidity and mortality seen in malnourished children. In a recent cohort study in almost 2,500 Indian children we demonstrated that those with poor folate status had higher incidence of diarrhea and pneumonia compared to those with normal folate status. This study also showed that that not being breastfed was associated with poor folate status. Moreover, the results from the study also indicate that the preventive effect of breastfeeding on respiratory and gastrointestinal infections partly can be explained by the folate content in the breast milk. This finding is new and needs to be verified in properly conducted clinical trials to establish a casual relationship. These trials should be carried out in populations where folate deficiency is prevalent such as the population selected for this study.

Hypothesis: Daily supplementation of 2 recommended daily allowances (RDA) of folic acid, with or without concomitant supplementation of 2 RDA of vitamin B12, in North Indian children 6 to 30 months of age, reduces the incidence of acute lower respiratory infections (ALRI), clinical pneumonia and diarrhea.

Design/Methods In a factorial design, we will conduct a randomized placebo controlled clinical trial of folic acid and vitamin B12 supplementation in 1000 children aged 6 to 30 months and living in a low to middle-income socioeconomic setting in New Delhi. Eligible children will be identified through a house-to-house survey. We will include between 4 and 5 children every day and they will be randomized to receive 2 RDA of vitamin B12 and folic acid, only folic acid, only vitamin B12 or placebo every day for 6 months.

Data We will compare the incidence of ALRI, clinical pneumonia and diarrhea between the treatment arms. This factorial design enables us to compare the effect of folic acid in children that are given B12 and in children that are not. The latter analysis is important, as utilization of folates requires appropriate vitamin B12 status. Furthermore, this design also enables us to measure the effect of daily vitamin B12 supplementation on childhood morbidity in this predominantly vegetarian population where vitamin B12 deficicency is extremely common.

Goal We seek to enhance child health by identification of nutrients that are related to diarrhea and pneumonia. If administration of these nutrients reduces the incidence of ALRI or diarrhea it can again result in new and inexpensive interventions that can reduce the burden of diarrhea and pneumonia in young children of developing countries.

Principal Investigator Tor A Strand

Hypothesis(es) and Aims- State concisely the hypothesis(es) to be tested and the specific aim(s) of the project. **Do not exceed one page.**

A. Hypothesis(es) to be Tested

Administration of 2 RDA of folic acid given with or without 2 RDA of vitamin B12 reduces the incidence of diarrhea, clinical pnuemonia and acute lower respiratory tract infections in children aged 6 to 30 months in a low to middle-income socioeconomic setting in New Delhi, India.

(RDAs from Food and Nutrition Board, Institute of Medicine, National Academy of Sciences, 2002).

B. Specific Aim(s) of the Project

1) To investigate whether daily supplementation of 2 RDAs of folic acid and/or 2 RDAs of vitamin B12 given to children 6 to 30 months of age can lessen
   a. the incidence of acute lower respiratory tract infections, and
   b. the incidence of diarrhea defined by three or more loose or watery stools and a recent change in consistency of stools

Secondary goals:
In North Indian children aged 6 to 30 months, measure the impact of folic acid and vitamin B12 supplementation;

1) on plasma levels of folate and vitamin B12,
2) on hemoglobin concentration and the risk of anemia,
3) on the incidence of clinical pneumonia as diagnosed by a medical doctor,
4) on growth, and
5) on the incidence density of febrile days.
6) Measure the impact of folic acid and vitamin B12 supplementation on end study developmental milestones.

And

7) the possible interaction between folic acid and vitamin B12 supplementation on the incidence of ALRI and diarrhea,
8) the possible interaction between folic acid and vitamin B12 supplementation on plasma levels of folate and vitamin B12, and
9) the prevalence of folate and vitamin B12 deficiency at baseline, and how baseline folate and vitamin B12 status affects trial outcome.
10) To measure the association between pneumonia incidence and the serum MBL concentration
11) To evaluate the exposure to *Cryptosporidium* spp. In Indian children aged 6-30 months over a six month period by measurement of antibodies to recombinant gp15, a conserved surface protein

12) To measure the association between the antibody response to *Cryptosporidium* and serum MBL, to determine the association between MBL concentration and *Cryptosporidium* infection.
13) To compare the change in serum mannose binding lectin (MBL) between those who have been given 2 RDA of vitamin B12 and/or folic acid with those who were given placebo
14) Estimate the vitamin D status in the study population and measure to what extent vitamin D Status predicts diarrhea or respiratory infections.

Provided additional funding we will also consider to measure;

1) the impact of folic acid and vitamin B12 supplementation on plasma levels of methylmalonic acid and total homocysteine

Principal Investigator: Tor A Strand

Background and Significance

Summarize what is already known about this child medical problem and the research gaps the project will attempt to address. Describe the potential contributions of the research to children's health. Include an estimate of how long it might take for these findings to become clinically relevant. **Do not exceed one page.** Include literature references in the Literature References page provided.

In children of developing countries, acute lower respiratory infections and diarrhea are still among the most common causes of death, claiming about 4 million lives every year (1). Known risk factors are young age, low birth weight, pollutants, poverty, malnutrition, zinc deficiency, HIV infection, and lack of breastfeeding (2). Therapeutic or prophylactic administration of zinc to young children reduces the risk of diarrhea and acute lower respiratory infections as well as episode duration (3-6). But other micronutrients may be equally important.

Folates play a crucial role in DNA and protein synthesis, suggesting that processes in which cell proliferation is essential may be impaired by poor folate status. Indeed, macroscopic disruption of the epithelial linings occurs with anti-folate treatment (7) and several facets of the immune system are affected by folate deficiency (7-9). The phagocytic and bactericidal ability of polymorphonuclear leukocytes is poor in individuals with severe folate deficiency and improves with folate replenishment (10-12). Furthermore, the thymus and cell-mediated immunity, the blastogenic response of T lymphocytes to certain mitogens and the antibody responses to several antigens is reduced in folate-deficient individuals (7). These changes to the immune system caused by folate deficiency may result in an increased susceptibility to infections. Indeed, individuals on antifolate treatment such as methotrexate have an increased risk of both mild and serious infections (13). Furthermore, poor folate status is associated with increased risk of pneumonia in older people (14). So far, however, little emphasis has been put on folate deficiency as a public health problem in children of developing countries. This is because a large proportion of mothers breast-feed their children for a long period of time and because many but far from all complementary foods contain relatively high folate concentrations(8, 15).

Breastfeeding, folate status and the risk of infections in children. The benefits of breastfeeding on child health are well documented. Breastfeeding prevents respiratory and gastrointestinal infections, even when continued up to the child completes his/her second year of life (16). Breast milk is rich in several nutrients including folates (17). Plasma folate reflects recent intake. Infants that are not breastfed have lower plasma folate concentrations compared to breastfed children of the same age. In a prospective study of 2,500, 6 – 30 months old North Indian children whom we followed for 4 months with weekly home visits (3, 18-20), we showed that children with low plasma folate concentrations had substantially and significantly higher risk of subsequently acquiring pneumonia and diarrhea as compared to children with normal plasma folate levels. This increased risk was independent of the status of several other nutrients (18). In the same study, we also demonstrated that breastfeeding reduced the incidence of pneumonia and diarrhea. Even more importantly, the association between breastfeeding and disease was substantially and significantly reduced when the plasma folate concentration was taken into account. In conclusion, having poor folate status seems to increase the risk of common infections and the effect of breastfeeding may to a large extent be explained by the folate provided by breast milk.

Thus, the efficacy of folic acid supplementation on childhood morbidity needs to be measured in rigidly conducted randomized trials in appropriate populations. We therefore want to undertake a trial on folic acid supplementation where we previously have seen a strong association between poor folate status and subsequent diarrheal and respiratory morbidity. Because vitamin B12 is required for proper utilization of folates (21-23), we will undertake a 2x2 factorial design where half of the folic acid recipients will also receive vitamin B12 and the other half will not. Similarly, half of those who do **not** receive folic acid will receive vitamin B12 while the other half will not.

Folic acid is cheap and non-toxic and, if our hypothesis derived from the above-mentioned cohort study in India can be verified, folate given in adequate amounts may counteract some of the negative consequences faced by children that are at risk of folate deficiency or those that cannot be breastfed, such as orphans or children of HIV infected mothers.

Mannose-binding lectin (MBL) When the adaptive immune response is either immature or compromised, the innate immune system is the principle defense against infection. MBL is a C-type serum lectin that binds microbial surface carbohydrates and mediates opsonophagocytosis directly by activation of the lectin complement pathway. A wide variety of clinical isolates of bacteria, fungi, viruses, and parasites are bound by MBL. Three polymorphisms in a structural gene are commonly found and result in production of low serum levels of MBL. Clinical studies have shown that MBL insufficiency is associated with bacterial infection in patients with neutropenia and sepsis, and that these effects are more pronounced in younger children and in bacterial pneumonias. Serum MBL levels have not been previously measured in children in India, and this study offers a unique opportunity to measure MBL levels at baseline in a cohort of children and then follow them for morbidity, mainly pneumonia and diarrhoea.

Most community-acquired pneumonia in young children will be caused by common bacterial pathogens and viruses. Since the etiology of pneumonias is diverse, we will not attempt to establish etiology, but will measure the association between MBL levels with subsequent disease. In addition to this aim, we will also study exposure and susceptibility to a specific pathogen, for which an association with MBL levels has been reported recently.

Cryptosporidiosis. Cryptosporidiosis is an important cause of diarrheal disease worldwide, particularly in developing countries. In these countries, malnourished children are at higher risk of being infected with *Cryptosporidium (Crypto)*, and *Crypto* infections in childhood have been shown to further compromise nutritional status and lead to impaired growth and cognitive function. The immune status of the host plays a critical role in determining the severity of cryptosporidiosis. Infection is self-limited in the immunocompetent hosts, but can be severe and persistent in the immunocompromised such as AIDS patients or malnourished children. Surveillance of enteric infections in India, has demonstrated high rates of symptomatic cryptosporidiosis in children, with *Crypto* the commonest cause of parasitic diarrhea in most settings.

Thus, cryptosporidiosis is more common and its consequences are more severe in malnourished than well-nourished children. In addition, children with cryptosporidiosis are at risk of further nutritional insults during the course of the infection. Studies of Haitian children reported that malnourished children with cryptosporidiosis have increased levels of systemic and fecal proinflammatory cytokines and lower levels of MBL. A recent report from Bangladesh has shown that serum MBL deficiency and polymorphisms in the -221 promoter region, were strongly associated with *Crypto* infections but the mechanism by which MBL mediates protection is unknown. MBL has been shown to bind to *Crypto* sporozoites, and may act by preventing infection of enterocytes. MBL is proposed to serve a more clinically important role in young children than in adults, especially during the vulnerable months after weaning (age 6–18 months) and before the adaptive immune system has matured.

Among the *Crypto* antigens implicated in mediating infection, the *Crypto Cpgp40/15* gene and its products gp40 and gp15 are the most important and antibody responses to the relatively conserved, immunodominant gp15 peptide have been associated with protection from diarrheal symptoms in *Crypto*-infected humans. Based on these data, we hypothesize that increased MBL levels will be associated with decreased infection and therefore, lower concentrations of antibodies to *Crypto*.

Cognitive functioning and folic acid and vitamin B12: Vitamin B12 deficiency is associated with cognitive deficits in the elderly (24). Although fewer studies, the association between cognitive functioning

and vitamin B12 status has also been described in children. Neonatal vitamin B12 deficiency causes failure to thrive and neurological manifestations, which may become irreversible (25). A study in the Netherlands showed that infants of macrobiotic mothers had delayed motor and language development compared to infants of omnivores (26). At age 12 years, the children had still higher MMA levels and scored lower than the omnivores on cognitive tests (27). Another study was conducted among Guatemalan school-age children. Children with vitamin B12 deficiency had slower reaction time on neuropsychological tests of perception, memory and reasoning, along with academic problems including lower academic performance, lower teacher ratings, more attention problems and more delinquent behavior (28). Furthermore, in a cohort of young Nepalese children, intake of meat and other animal products, which are good sources of vitamin B12 was positively associated with cognitive performance (29). In a cohort study in North India we demonstrated that children with poor vitamin B12 status (i.e. low plasma vitamin B12, elevated MMA acid or homocysteine) had lower mental development scores on the Bayley Scales of Infant Development II (BISD II) than those that did not have signs of vitamin B12 deficiency. These associations remained strong and significant even after adjusting for potential confounders such as socioecomomic status, maternal and paternal education, nutritional status, age, sex, among others. If we are able to demonstrate a causal relationship between vitamin B12 and/or folate and cognitive functioning in children in low-income countries, this will probably influence national nutrition recommendations and the recommended composition of complementary foods.

Vitamin D deficiency is common worldwide and has been described in India (30, 31) (32). The main source of vitamin D is sunlight. Vitamin D deficiency is associated with immune function and risk of respiratory tract infections (33, 34). Data from young children in low and middle-income countries is, however limited. In another large, randomized clinical trial we recently observed that, for a short period after the cold and rainy season, several children had convulsions. Hypocalcaemia secondary to poor vitamin D status could explain this. However, we could not measure vitamin D status and data from this population is limited. In this study we collect plasma from all included children. Analyzing vitamin D in these children will give us an excellent opportunity to describe vitamin D status in this population, across various seasons and in different age categories. Furthermore, the cohort design enables us to measure to what extent vitamin D status is associated with diarrhea and respiratory tract infections.

Principal Investigator Tor A Strand

Supportive Preliminary Data

Include research conducted by the investigator(s) that leads to the present proposed research project. **Do not exceed one page.** Include literature references in the Literature References page provided.

The cohort study mentioned in the previous section generated the hypothesis behind this proposal. In this study we observed 1,176 episodes of acute lower respiratory infections in 2,500 children. Mean vitamin B12 concentration was low and mean MMA concentration was high indicating that vitamin B12 deficiency was very common (Table 1). B12 status, however was not associated with increased risk of pneumonia or diarrhea (18, 19).

This cohort study also revealed that poor folate status was more common than anticipated and that children that were not breastfed had poor folate status. In fact, breastfed infants had almost a four-fold higher plasma folate concentration than did those that were not breastfed (Table 1). The concentration of the markers of folate and cobalamin (vitamin B12) in various breastfeeding and age categories are shown below (18, 19).

Table 1: plasma concentrations of folate, cobalamin (vitamin B12), methylmalonic acid (marker for vitamin B12), and total homocysteine (marker for folate and vitamin B12) in young Indian children. Table indicates high prevalence of vitamin B12 and folate deficiency.

	**Breastfed**				**Not breastfed**			
	**6-11 months**		**12-30 months**		**6-11 months**		**12-30 months**	
**Median and IQR**								
Cobalamin, pmol/L [2]	184	120 - 263	172	124 - 253	334	235 - 463	261	194 - 348
Folate, nmol/L [2]	20.2	11.7 - 34.4	11.3	7.4 - 17.6	5.3	3.4 - 7.7	6.5	4.7 - 9.2
Total homocysteine µmol/L	12.6	9.2 - 18.1	11.3	8.7 - 15.2	10.7	8.2 - 13.9	9.1	7.4 - 11.2
Methylmalonic acid µmol/L	1.03	0.54 - 2.08	0.74	0.42 - 1.36	0.45	0.31 - 0.71	0.38	0.26 - 0.59

Being in the lowest quartile of folate status increased the risk of pneumonia during the subsequent 4 months by 44% (18) and of diarrhea by 50 % (unpublished results). These estimates of increased susceptibility were adjusted for other risk factors for childhood illness such as stunting and wasting, age, socioeconomic status, crowding, season, and breastfeeding status. Having elevated homosysteine, which is an unspecific marker of folate and vitamin B12 status, did also increase the risk for pneumonia and diarrhea during the following four months.

Our study showed that vitamin B12 deficiency was prevalent but not associated with disease. However, clinical vitamin B12 deficiency is also associated with histochemical alterations in the intestinal mucosa and increased risk diarrhea. The 2x2 factorial design of this clinical trial (figure 1 and 2) enables assessment of the effect of vitamin B12 administration on diarrhea and acute lower respiratory infections .

Combined supplementation with iron and folic acid in children in Zanzibar was associated with increased risk of severe infections and deaths (35). It was assumed that iron caused this effect and that the increased risk of severe infections was due to malaria although this was not confirmed. However, folic acid could still be the cause and it is well known that folate interferes with some antimalaria agents (36). The first line antimalarial treatment in the trial mentioned above was an anti-folate combination of sulfadoxine and pyrimethaminthe. When folic acid and iron has been given with this drug, there has been substantial delay in parasite clearance (37). Thus, the combination of folic acid and iron has been associated with increased susceptibility to childhood infections. We still do not know which of the nutrients that caused this effect in increased morbidity. It is accordingly crucial to investigate the efficacy of folic acid alone (without iron) on common and potentially dangerous childhood illnesses such as pneumonia and diarrhea.

Experimental Design and Methodology

Discuss *in detail* the design and procedures to be used to accomplish each specific aim of the project. Describe the protocols to be used, and provide a tentative sequence or timetable for the project. Include a description of facilities and other resources and the means by which you would analyze and interpret the data. If any new methodology is to be implemented, outline its expected advantage over existing methodologies. Discuss the potential difficulties and limitations of the proposed procedures and alternative approaches to achieve the aims. Point out any procedures, situations, or materials that may be hazardous to personnel and the precautions to be taken to prevent any harm. Be complete but focused in your presentation and provide sufficient detail in the narrative to allow peer reviewers to make valid judgments regarding the quality of the project proposal. **Do not exceed ten (10) pages**, excluding references. Include literature references in the Literature References page provided.

Design: Factorial design. Randomized double-blind placebo-controlled preventive field trial.

Choice of approach and methodologies. The impact of supplementation with folic acid and/or vitamin B12 will be measured using a randomized double-blind placebo-controlled study design. Placebo, folic acid and or vitamin B12 supplementation will be maintained at home for 26 weeks.

Figure 1, study design

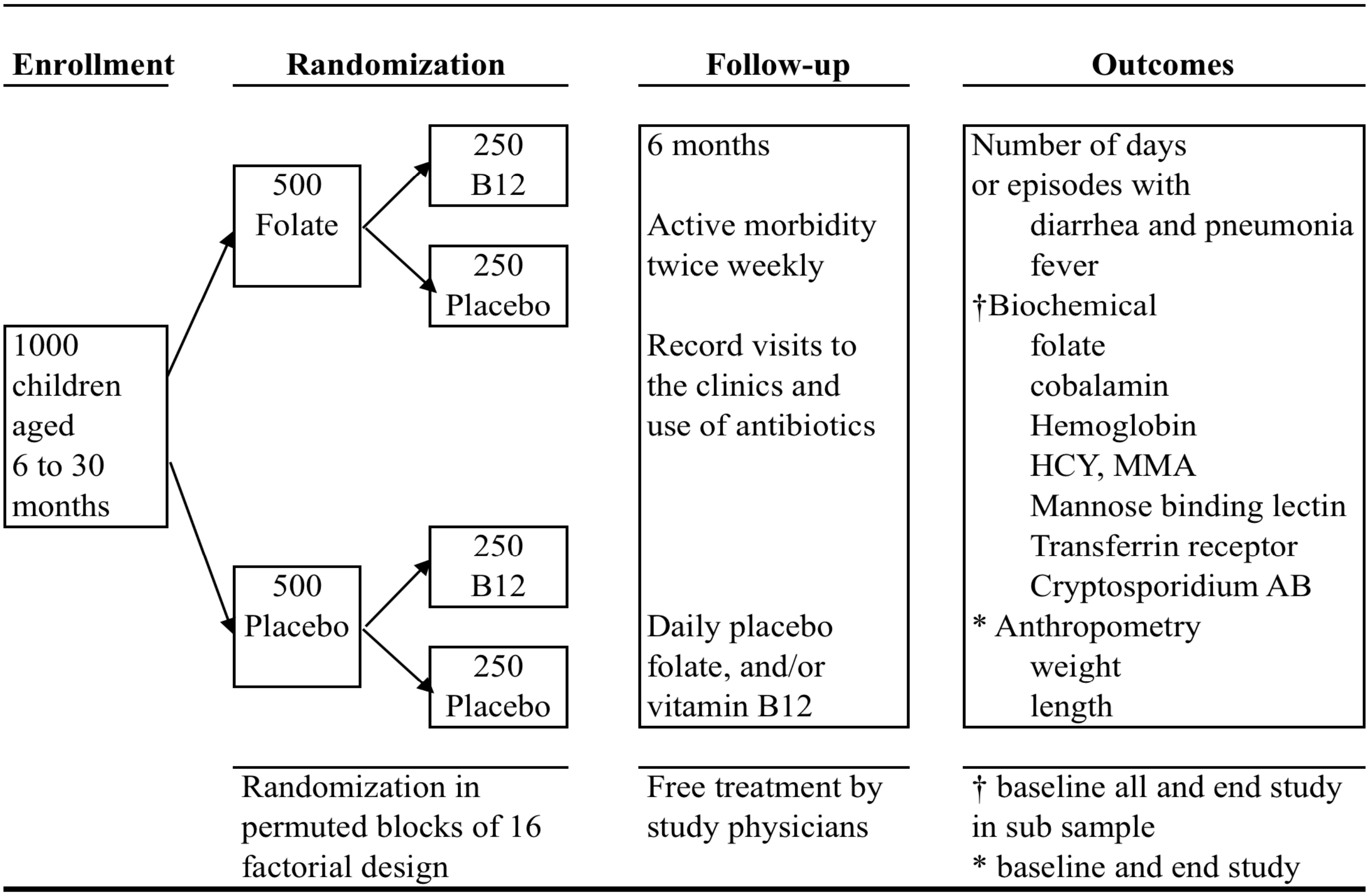

anemic children will also be given iron according to IMNCI guidelines

IMNCI: Integrated management of neonatal and childhood illness (see below)

Study design and methods

The study will be conducted in the low to middle socioeconomic settings of Tigri and Dakshinpuri in New Delhi with a total population of about 300,000. We have established a confident interaction with the study

population. Furthermore, there is already an intact infrastructure, including a health care delivery system as well as a morbidity surveillance scheme with a dedicated staff geared towards the envisaged activities. The health services are provided through health care centres. The investigators have carried out a number of research projects at this site. Among the adult population, 80% of the females and 20% of the males have never been to school, 50% of under-fives are stunted (height for age < -2z), the incidence of diarrhea in the age group <30 months is 7 episodes/child/year. About 10% of these episodes are persistent, contributing to ≈50% of all diarrhea-related deaths.
The prevalence of WHO defined pneumonia and clinical pneumonia is 1.4 episodes and 0.32 episodes per child year, respectively. Other relevant features of this population (from our most recent study) are described in the table below:

Selected features of the target population.

			SD or (IQR)
Median years of school of mothers (years)		5	(0, 9)
Median years of school of fathers (years)		9	(7, 10)
Median household income (in 1000 rupees)		36	(24, 54)
Proportion of families that are multigenerational		48%	
Z score weight for age		-2.0	1.01
	<-2	50.0%	
	<-3	14.0%	
Z score weight for length		-1.15	0.92
	<-2	16.9%	
	<-3	1.1%	
Z score length for age		-1.66	1.09
	<-2	35.2%	
	<-3	1.0%	

Criteria for inclusion of children. Children will be enrolled if they fulfill the following inclusion criteria:
*Age 6-30 months
*Availability of informed written consent
* Plan to reside in the area for the next six months
* Either sex

Criteria for exclusion of children.
- Severe systemic illness requiring hospitalization
- Severe malnutrition, i.e. weight for height < -3 z of the WHO standard for this age group. For ethical reasons these children require micronutrient supplementation and adequate medical care.
- Lack of consent
- Taking B vitamin supplements that include folic acid and vitamin B12.
- Severe anemia (Hb < 7 g/dL). This would be a temporary exclusion and the children will be enrolled if this is successfully treated.
- Ongoing acute infection with fever or infection that requires medical treatment. This would be a temporary exclusion and the children will be enrolled after recovery.

**Table 2. DEFINITIONS** (38)
*Diarrhea: Passage of ≥ 3 loose or watery stools/24 h period<br>*Children are considered to have recovered from a diarrheal episode on the first of two consecutive diarrhea free days<br>*Prolonged diarrhea: Diarrhea of ≥7 days' duration with no more than 2 intervening diarrhea free days<br>*Persistent diarrhea: Diarrhea of ≥14 days' duration with no more than 2 intervening diarrhea free days<br>*Severe diarrhea: Passage of ≥5 liquid stools on at least one day of the episode<br>*ALRI= Acute lower respiratory infections:<br>Emic-WHO criteria: Cough + mothers report of fast or difficult breathing, in local language/terminology<br>Clinical criteria: Cough + elevated respiratory rate above age-specific cut-off values according to WHO-criteria<br>ALRI: Cough and fast breathing or lower chest indrawing as assessed by the physician<br>*Clinical Pneumonia: Diagnosed either by a combination of cough with crepitations or bronchial breathing by auscultations or as an episode of acute lower respiratory tract infection associated with at least one of lower chest indrawing, convulsions, not able to drink or feed, extreme lethargy, restlessness or irritability, nasal flaring or child is abnormally sleepy and difficult to wake<br>* Fast breathing: ≥50 breaths per minute in infants and ≥40 breaths per minute in older children.

Random allocation to intervention groups and blinding procedures. Eligible children will be allocated by block randomization to one of the intervention groups outlined in **figure 1**. The block randomization procedure compensates for possible seasonal variation in disease incidence as the block length controls for seasonality ensuring against unequal number of allocations even over short time intervals. Furthermore, the block randomization procedure enables smaller sub studies within the main trial where equal numbers of participants in each of the four treatment arm is secured. Placebo and supplements will be similar in appearance and taste. The supplements/placebos will be offered to the enrolled children according to serial numbers provided by the producer. The randomization will be stratified into infants and other children. This will be done by assigning blocks to either of these two strata.

Clinical parameters. All children will be visited twice weekly to obtain morbidity data. At the visits, mothers will be asked about diarrheal illness (number and consistency of stools), symptoms of respiratory illness (cough, fast or difficult breathing), fever and physician visits **(Table 3)**. Respiratory rates will be measured at each visit. Weight and length/height will be measured at enrollment and at the end of the 26-week observation period. Developmental milestones will be measured at the end of the 26 week observation period. Sick children that might require treatment will be referred to the study clinic for treatment. The threshold for such referral is low to ensure proper sensitivity. The specificity of the diagnosis will be ensured by examination of the study doctor.

Laboratory parameters. Blood samples will be obtained at enrollment (base-line) for all children and at the end of the 26 week follow-up period in a randomly selected subsample of 256 children (16 blocks). Three mL of blood will be collected into vacationers containing EDTA. The plasma will be centrifuged at approx 700 g at room temperature for 10 minutes, separated and transferred into storage vials, and stored at -70 degrees before analysis.

The blood samples will be analyzed for

1) Hemoglobin
    a. EDTA blood from will be analyzed for hemoglobin by HemoCue B-Hemoglobin.
    b. For children where blood from the cubital vein is not obtained, capillary blood will be used for analysis of Hemoglobin

2) Plasma cobalamin (vitamin B12), and plasma folate concentration will be estimated by microbiological assays using a chloramphenicol resistant strain of *Lactobacillus casei* and colistin sulphate resistant strain of *Lactobacillus leichmannii*, respectively (39, 40).
3) Plasma transferrin receptor (marker of iron status) and
4) Plasma methylmalonic acid and total homocysteine will be analyzed by a modified gas chromatography-mass spectrometry method based on ethylchloroformate derivatizations
5) Serum MBL levels will be determined by enzyme-linked immunosorbent assay (ELISA) using the Human MBL (Lectin Assay) ELISA test kit (HyCult Biotechnology). This solid-phase ELISA uses mannan-coated plates to capture functional MBL. MBL levels will be calculated by means of a standard curve based on samples of known MBL concentration.
6) The concentration of anti Cryptosporidium antibodies will be measured using standard ELISA
7) Vitamin D (25 OH vitamin D) will be measured for all children at baseline using standard methods.

<u>**Measurements.**</u>

***Anthropometry.** Weights will be measured with a portable salter scale that measures to the nearest 25 g (portable Salter). Height and length will be measured according to standard guidelines, through locally made infantometer.

***Developmental milestones.** The developmental milestones will be measured using the ASQ-3. ASQ-3 is an easily administered and comprehensive checklist consisting of 30 items measuring skills in 5 different domains; Communication, Gross Motor, Fine Motor, Personal-Social and Problem-Solving. The questionnaires are divided into two-month intervals for use with children 4-60 months of age, and scores are normed to indicate whether children are developing age-appropriately.

Intervention:

The intervention will be provided in plastic 330g jars containing supplement for one month. 6 jars are needed for each child but we will include 2 extra for each enrolled child in case of loss.

The composition of the lipid based nutritional supplement will be as follows

1) Placebo
2) 2 RDA of folic acid,
3) 2 RDA of vitamin B12, or
4) 2 RDA of vitamin B12 and 2 RDA with folic acid

	**Placebo**	**Vit. B12**	**Folate**	**Vit. B12 + Folate**
Nutriset Code	C30-18	C30-20	C30-21	C30-22
Daily ration (g)	10g	10g	10g	10g
Type of packaging	330g jar	330g jar	330g jar	330g jar
Total energy (kcal)	54.1	54.1	54.1	54.1
Protein (g)	0.71	0.71	0.71	0.71
Fat (g)	3.31	3.31	3.31	3.31
Folic acid (µg)	-	-	150	150
Vitamin B12 (µg)	-	1.8	-	1.8

Iron (mg)*	0.06	0.06	0.06	0.06
Zinc (mg) *	0.13	0.13	0.13	0.13
Cu (mg)*	0.02	0.02	0.02	0.02
Calcium (mg)*	13.43	13.43	13.43	13.43
Phosphorus (mg)*	15.15	15.15	15.15	15.15
Potassium (mg)*	55.38	55.38	55.38	55.38
Magnesium (mg)*	7.14	7.14	7.14	7.14

* provided by ingredients (Peanut, Mix of vegetable oil, Sugar, Maltodextrine, DSM, Cacao).

The supplement will be prepared by NUTRISET, Ltd (MALAUNAY, France). Staff at NUTRISET will label the jars and the code that links the serial number of the packages to the group identity will be kept with NUTRISET and with a statistician otherwise not involved in the trial. The researchers involved in this trial will not get this list until the analysis stage (see below).

We will give 2 RDA of folic acid

The RDA for dietary folate equivalents (DFEs) for children 1-3 years of age is 150 micrograms per day (Food and Nutrition Board, Institute of Medicine, National Academy of Sciences, 2002). The RDA is expressed as DFEs because synthetic folic acid is more easily absorbed in the intestine than naturally occurring folate. One microgram of naturally occurring food folate is equivalent to 0.6 micrograms of folic acid from fortified foods or supplements consumed with meals and to 0.5 microgram of supplements not consumed with meals. Thus one RDA corresponds to 75 µg (0.5 x 150) of synthetic folic acid given as a supplement and 2 RDA of folic acid to this group is 150 micrograms per day. In 6 to 11 month old children the RDA for folate is 80 micrograms.

We will give 2 RDA of vitamin B12

The RDA for vitamin B12 in children 1 – 3 years of age is 0.9 micrograms per day (Food and Nutrition Board, Institute of Medicine, National Academy of Sciences, 2002). I.e we will give the toddlers randomized to receive vitamin B12, 1.8 micrograms B12 per day. In 6 to 11 month old children the RDA for B12 is 0.5 micrograms.

This amount of folic acid and vitamin B12 for six months does not constitute any health risk for the child. However, we will closely monitor possible adverse effects during follow-up. This will be done by assessing morbidity by the morbidity fieldworker and by observing the child for 20 minutes after the placebo, folic acid and/or vitamin B12 was given.

Co-intervention.

All caretakers will be given dietary recommendations according to national guidelines.

Children with anemia (defined according to IMNCI guidelines) will be given peroral iron.

IMNCI guidelines are available from Ministry of Health and Family Welfare, Government of India. (Integrated management of neonatal and childhood illness: Physician chart booklet.) Accessible at http://mohfw.nic.in/dofw%20website/training_material_for_imnci_for_Phy.htm)

In short:

At enrollment (all) and end study (in subsample) when we will measure Hemoglobin concentration (Hb) in all children.

Children with Hb 7 – 10.9 g/dL will be treated with peroral iron + folic acid for 14 days and included.
Children with Hb < 7 g/dL will not be enrolled and referred to hospital

When enrolled (i.e. during the period when receiving placebo, folic acid and/or B12):
Children with palmar pallor will be treated with peroral iron + folic acid for 14 days.
In children with severe palmar pallor we will measure Hb.

Children with Hb 7 – 10.9 g/dL will be treated with peroral iron for 14 days (or longer if still anemic).

Children with Hb < 7 g/dL will be referred to hospital.

Treatment

Children with pneumonia and dysentery will be treated according to IMNCI guidelines.

Table 3. KEY OUTCOME MEASURES FOR COMPARISON BETWEEN STUDY GROUPS, MEASURES OF IMPLEMENTATION EFFECTIVENESS AND BIOCHEMICAL MARKERS	
**Impact on morbidity and growth**<br>• Incidence of all diarrhea<br>• Incidence of severe diarrhea<br>• Incidence of prolonged diarrhea (≥7d)<br>• Incidence of persistent diarrhea (≥14d)<br>• Diarrhea prevalence:<br>Mean daily diarrhea prevalence<br>Mean daily severe diarrhea prevalence<br>• Incidence of ALRI<br>• Incidence of clinical pneumonia<br>• Physician visits for diarrhea and for ALRI<br>• Mean % change in weight and length/height<br>• Mean z-scores: height for age and weight for length/height<br>• Days with fever<br>• Hospital stay<br>• Deaths<br>• Adverse events (vomiting, gastric discomfort)	**Establishing whether folic acid and vitamin B12 delivery by field workers was implemented effectively**<br>Plasma folate and vitamin B12 at the end of the 26 week follow-up.

Data management, sample size justification, and analyses.

Comparisons

Figure 2: The four treatment arms

	**Folic acid** -	**Folic acid** +
**B12** -	A<br>placebo only<br>N=250	B<br>folic acid only<br>N=250
**B12** +	C<br>B12 only<br>N=250	D<br>folic acid & B12<br>N=250

**Table 4. Treatment groups that will be compared.**<br>**Main comparisons are as follows (referring to figure 2)**	**Comparisons**	**Sample size for this analysis †**
**1) Folic acid vs placebo folic acid**	**A and C vs B and D**	**1000**
**2) Vitamin B12 vs. placebo B12**	**A and B vs Cand D**	**1000**
**3) Folic acid vs. placebo folic acid and not daily B12**	**A vs B**	**500**
**4) Folic acid vs. placebo folic acid and daily B12**	**C vs D**	**500**
**5) Vitamin B12 vs. placebo and not daily folic acid**	**A vs C**	**500**
**6) Vitamin B12 vs. placebo and daily folic acid**	**B vs D**	**500**

† total sample size, i.e. the group size is half of these figures.

Sample size calculations

These calculations are based on earlier studies on the efficacy of routine administration of zinc that have been carried out in the same population. These calculations assume a probability of a Type I error of 5% and a Type II error of 10% and 20% (power of 90% and 80%, respectively). In order to detect a 25% reduction in the incidence of ALRI in the community, we need a sample size of 420 per group. Allowing for up to 10% attrition and adding another 10% in order to adjust for possible clustering, we need to recruit 1000 children (500 per group) to compare the effect of folate vs placebo on ALRI incidence with 90% power. This calculation assumes that the efficacy of folate is similar among children who receive vitamin 12 and those who do not (i.e. no interaction between folic acid and vitamin B12 administration).

We have not calculated the sample size for the interactions between folic acid and vitamin B12 on any of the outcomes. The main focus of this study is to assess the efficacy of folic acid administration and or vitamin B12 administration. We have sufficient power to undertake these comparisons for several of the outcomes even when comparing only two of the four cells in figure 3 (i.e. comparisons 3-6 in table 4). The interaction between folic acid and vitamin B12 will be assessed for all of the outcomes mentioned in table 3.

The sample sizes required for the relevant outcomes are displayed in the table below:

Table 5. Estimated sample size requirements for various outcomes (incidence reductions) and power*

	Episodes per child year			Required sample size / 6mo with a power of:	
**Outcome**	**Placebo**	**Intervention**	**% reduction**	**90%**	**80%**
ALRI	1.4	1.05	25%	420	314
	1.4	0.98	30%	283	212
	1.4	0.91	35%	202	151
	1.4	0.84	40%	150	112
	1.4	0.77	45%	115	86
	1.4	0.70	50%	90	67
Clinical pneumonia	0.32	0.19	40%	667	498
	0.32	0.17	45%	510	381
	0.32	0.16	50%	400	299

Diarrhea	6.00	5.10	15%	288	215
	6.00	4.50	25%	98	73
	6.00	4.20	30%	66	49
	6.00	3.90	35%	47	35
Prolonged diarrhea > 7 days	1.00	0.75	25%	588	439
	1.00	0.70	30%	397	296
	1.00	0.65	35%	283	211
	1.00	0.60	40%	210	157
	1.00	0.55	45%	161	120
Severe diarrhea (any day with stool frequency of > 9)	0.90	0.63	30%	441	329
	0.90	0.59	35%	314	235
	0.90	0.54	40%	233	174
	0.90	0.50	45%	179	133
	0.90	0.45	50%	140	105

We will also draw an end study sample from 256 children which is 16 blocks (each block consist of 16 children 4 in each of the treatment arms). The blocks will be selected randomly. Selection of blocks rather than children ensures equal numbers of children from each of the four study groups. Allowing a 10 % loss to follow up and another 10% failed blood sampling, this sample size gives us a power of 90% to detect a 50% increase in the folate concentration and a 25% increase in the B12 concentration. These calculations assumes initial mean (SD) folate and cobalamin concentrations of 15 (14) and 240 (140) , respectively.

For the developmental outcomes we will try to reach a sample size of 400, 100 per group. For the sample size calculations we have assumed that the ASQ scores will be normally distributed, a mean score of 40 (SD 10) for all dimensions, and a minimum meaningful difference of 5 points. When assuming a power of 90% and an alpha error of 5%, the required sample size becomes 85. To allow for 15% attrition, we have arrived on a sample size of 100 per study group (comparisons 3-6, table 4). For comparison 1 and 2 in table 4, the power to detect this difference will be even higher.

Data management and analysis
All forms will be checked manually by supervisors for completeness and consistency. The data will then be double entered at the local sites into appropriate databases with computerized range and consistency checks. In this process there will be a continuous data cleaning. Comparison of baseline features including plasma folate and vitamin B12 levels will be made to confirm comparability across treatment groups.

We will use the statistical software packages Stata and SAS to analyze the data. A detailed plan for analysis of the main outcomes will be made before the list that links the child identity numbers to the treatment groups are obtained by the researchers. Data cleaning, definition of outcome variables, exclusion of cases as well as programming of scripts in the statistical packages will also be done before the analysis-files are merged with the randomization lists. The intervention groups will be coded in a way so that the researchers do not know the group identities until the main analyses are finished. In these analyses there will be two variables denoting the group identity, each of these two variables has to levels indicating whether the child was given folic acid or not or vitamin B12 or not. The analyses will be planned and undertaken in a joint workshop attended by the involved scientists. All analyses will initially be done on an intent-to-treat- basis, to adjust for potential baseline differences we will use generalized linear models with appropriate link functions (logistic, Poisson and/or negative binomial models). When analyzing comparison 1 and 2 (table 4), the interaction between folic acid and vitamin B12 will be tested in the regression models for all relevant outcomes.

Dissemination of the results

The results of this trial will be published in international biomedical journal with peer review. The Indian PI will be responsible for the manuscript describing the main outcomes of the trial. The PI in India is also responsible for rapid dissemination to local and national health authorities. This process will start as soon as possible after the meeting where the analysis workshop is held.

Risk of the project
There is a risk of misclassification of the outcomes in this project. Trained fieldworkers and doctors will make the diagnoses. The potential misclassification will be non-differential and will, if they occur, tend to shift our estimates toward a null effect. To avoid such misclassifications we will take the following measures:

1) The staff will go through extensive training before study start. Only those who reach a certain level of skills in diagnosing diarrhea and acute lower respiratory infections, will be allowed to work as morbidity fieldworkers. They will be assessed prior to study start and intraobserver and interobserver (comparison with a gold standard) variability will be measured to quantify their skills. Such evaluation of the staff will also take place on regular intervals throughout the trial.
2) Supervisors/Fieldworkers will be trained to screen developmental milestones checklist at the end of the 26 weeks observation period using the ASQ-3. Only those supervisors/fieldworkers who reach a certain level of skills in performing the checklist will be allowed to perform the screening.
3) A manual with the definitions and protocol will be kept in the field office and the field workers will at all times carry a short version of the manual describing their tasks.
4) Supervised (observation of the field workers at work) and non-supervised (assessment of the quality of the performance of the field worker after the field worker have made a home visit) will be undertaken in 5% of all household visits. These quality checks will be done by the supervisors and selected randomly among the enrolled children. On the non-supervised visits, the supervisors will ask questions on the behavior of the field workers and for the morbidity visits they will fill in a morbidity form that will be compared with that of the field worker. This system ensures that misclassification of the exposure is kept to a minimum by ensuring that dispensing is being done. It also ensures that the misclassification of the outcome is kept to a minimum by ensuring correct diagnosis.
5) Field workers will refer ill children to the clinic and doctors who are supervised by pediatricians will decide the diagnosis. The diagnosis made by the morbidity field workers ensures high sensitivity while the doctors ensure high specificity of the outcomes of interest.
6) There will be two doctors in the health clinic at any time that can consult each other if in doubt of the correct diagnosis.

Ethical considerations
Clearance from the medical ethics committee at SEHAT, SAS , CMC and the regional ethical committee of Norway that is responsible for university of Bergen (Regional Med Resch Ethics Comm West) will be obtained before the study is initiated. All aspects of the study will be in agreement with the latest version of the Helsinki declaration and we will undertake and report the study in accordance to the guidelines of the "Consort" statement (41).

Data safety monitoring board
A data safety monitoring board will be considered constituted for the study. The members will include a pediatrician, a statistician and a clinician with clinical trial experience. The members of this committee will not be involved in the study. They will undertake an interim analysis if they find this necessary.

Trial registration
The trial is registered in clinicaltrials.gov

Mitigation

During the project period, we will staff a field clinic where all eligible children will be offered free examination and treatment for common conditions including diarrhea and ALRI. Children will be treated for common infections according to IMNCI guidelines. Children with anemia will be offered free treatment with iron according to IMNCI guidelines (see above).

Implementation strategy

Inclusion criteria

- Age: 6 to 30 months
- Either sex
- Likely to reside in area for next 6 months
- Availability of informed consent

Exclusion criteria

- Severe systemic illness requiring hospitalization
- Severe malnutrition, i.e. weight for height < -3 z of the WHO standard for this age group. For ethical reasons these children require micronutrient supplementation and adequate medical care.
- Lack of consent
- Taking B vitamin supplements that include folic acid and vitamin B12.
- Severe anemia (Hb < 7 g/dL). This would be a temporary exclusion and the children will be enrolled if this is successfully treated.
- Ongoing acute infection with fever or infection that requires medical treatment. This would be a temporary exclusion and the children will be enrolled after recovery.

Figure 3: Study timeline

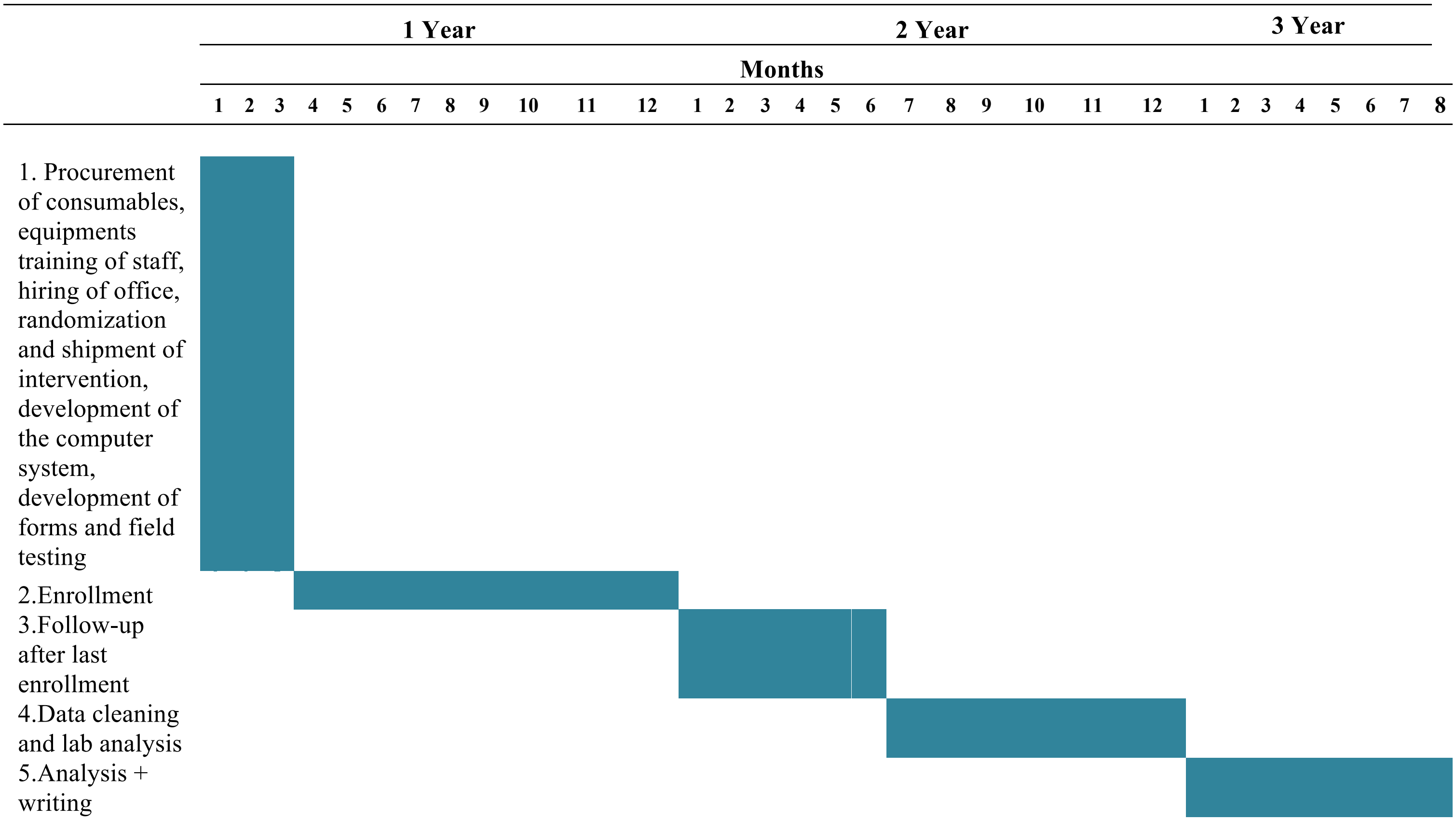

Table 6. Study Procédures

Activity	When	By Whom
Anthropometry (weight, length)	At enrollment, 6 months post enrollment (all children)	Field workers
Blood sample collection	At enrollment (all), 6 months post enrollment (250 children)	Physicians, Phlebotomist
Folic acid, Vitamin B12, Placebo supplementation	Daily supplementation (6 months)	Field worker; dispensing
Morbidity ascertainment	Biweekly throughout study period (6 months)	Field worker; morbidity ascertainment
Developmental milestones	6 months post enrollment (400 children)	Field workers
Referral to clinic	Whenever predetermined criteria are present on a morbidity visit	Field workers
Referral to physician for screening for clinical pneumonia	All cases having cough and high respiratory rates (≥6 months to <12 months RR ≥45, ≥12 months RR ≥35) or lower chest indrawings	Field workers

Activity	When	By Whom
Diagnosis of clinical pneumonia	Through all cases referred by Field worker and spontaneous visits to clinic	Physician
Diagnosis of other illnesses	Throughout study period	Physician
Spontaneous visits to clinic by mothers	Whenever they feel child needs to see a doctor	Physician

Table 7: Outcome measures

Activity	When	By Whom
Morbidity	Incidence of diarrhea, dysentery, persistent diarrhea<br>ALRI<br>By mothers definition<br>By WHO criteria<br>Diarrhea<br>By mother definition<br>By field workers definition<br>Dysentery<br>Persistent diarrhea<br>Days of febrile illness	Mother bring children spontaneously to clinic for illness<br>On biweekly visits made by field workers to homes throughout study period
	Clinical Pneumonia	Through all the cases of suspected ALRI referred by the field workers
Anthropometric status	Weight for age, weight for height, height for age at enrollment and after 6 months of supplementation (all children)	-
Biochemical assays	Haemoglobin, plasma folate, B12, Hcy (MMA, Transferrin receptor, Mannose binding lectin. Cryptosporidium AB) at enrollment (all children) and after 6 months of supplementation in 250 children	-
Developmental milestones	After 6 months of supplementation in 400 children.	

Survey
The study will be conducted in low to middle socioeconomic neighbourhoods of Dakshinpuri and Tigri. A door-to-door survey will be conducted to identify households with children aged 0 to 30 months.

Screening and Consent Procedures
Eligible and willing children aged 6-30 months will be brought to the clinic along with their caregivers. Here they will be screened by the physician/supervisor for eligibility criteria. Only one child (younger one) will be enrolled from each household.

Prior to enrollment, informed consent will be obtained by the physician/supervisor. An information sheet will be read out to the caregiver. The caregiver will sign the consent form; if the caregiver is non-literate, the consent will be witnessed by an impartial witness. A list of witnesses will be kept.

Prior to initiating supplementation, socioeconomic details of the enrolled child will be collected, and weight and length will be taken. The baseline blood sample will be collected. The first dose of supplement will be administered to the child. The supplement jar will be handed over to the mother.
Field workers in charge of supplement administration will visit the child's home daily on all working days and administer the supplement to the child. On Sunday, instructions will be left with the mother to dispense the same to the child. The supplements will be replaced by fresh jar at home by the field worker fortnightly. If the mother is travelling, the days she is not available apaques sachets will be prepared and given by field workers.

Another set of field workers will visit the child's home biweekly for morbidity ascertainment for a period of 6 months. At each visit, information will be obtained on a day wise basis for all the days elapsed since the previous visit on whether the child had any illness(es) such as fever, diarrhea, respiratory morbidity etc. At each visit, the field worker will refer patients to the study clinic if they possess any of the indications indicated in the predetermined referral criteria.

Morbidity visits
Trained fieldworkers will visit the households two times every week. During these visits they will ask for day vice symptoms of illnesses, morbidity, visits to other treatment facilities, hospitalizations for the past 3 or 4 days. The fieldworkers will also undertake a physical examination of the children that includes assessment of dehydration (if the child has diarrhea), respiratory rate count (if the child has symptoms of lower respiratory illness), measure temperature (if caretaker reports fever).

Referral of sick children
The study sites are urban resettlement neighborhoods where access to transportation (buses, three-wheelers, taxis) is available at all times of the day and night. Several tertiary hospitals are located within 3-7 kms of the study setting. Some major ones are Batra Hospital, Apollo Hospital and the All India Institute of Medical Sciences. The latter is a public hospital where inpatient and outpatient services are provided free of cost. Additionally, clinics run by the government (that treat free of cost) and private practitioners (who charge a nominal sum of money) are located within these neighborhoods or at a short distance away.

At enrollment a participant card will be given to the family. This card will include the study clinic address and mobile phone numbers of the study coordinator and the Indian principal investigators. Families will be asked to visit the study clinic (between 8 am to 6 pm) or call any of the mobile phone numbers whenever their child is ill. Additionally, at all home visits children that fulfill referral criteria (boxed below) will be escorted to the study clinic. Whenever study physicians identify a need for hospitalization, the child will be transported to one of study hospitals mentioned above and costs incurred, if any, covered by the study.

A) Criteria for referral to the field clinic for examination by one of the study physicians

For the field workers

REFERRAL CRITERIA –Refer the infant to the study clinic if the infant has the following	
Not able to drink<br>Vomits everything<br>Convulsions<br>Lethargic or unconscious<br>Abnormally sleepy or difficult to wake<br>Lower chest indrawing<br>Stridor in a calm child<br>Fast breathing (≥ 50 breaths/minute in 6 months	Fever with neck stiffness<br>Hyperthermia i.e. axillary temperature > 41°C<br>Bulging fontanelle<br>Tender swelling behind the ear<br>Visible severe wasting<br>Oedema of both feet<br>Severe palmar pallor<br>Deep extensive mouth ulcers

to <12 months)<br>(≥ 40 breaths/minute in 12 months to 36 months)<br>Some dehydration<br>Severe dehydration<br>Diarrhea for 14 days or more	Clouding of cornea<br>Pus draining from ear<br>Measles<br>Severe malnutrition<br>Mother wants infant to be seen by physician

B) Criteria for acute referral to hospital for severe illnesses. These criteria are based on the Integrated Management of Neonatal and Childhood Illness (IMNCI) algorithms.

For the study physicians

1) Severe malnutrition

Visible severe wasting or,
Oedema of both feet

2) Severe anemia

When severe palmar pallor is present, we will measure hemoglobin and urgently refer to hospital if hemoglobin < 7 g/dL. If hemoglobin is over this cut-off, the child will be treated for iron-deficiency anemia at home and subsequently followed up as per national guidelines.

3) Severe pneumonia with any of these danger signs

Convulsions during the present illness,
Unconscious or lethargic,
Unable to drink or breastfeed,
Vomits everything he or she eats
Stridor in a calm child
Lower chest indrawing

4) Diarrhea

With severe dehydration (for intravenous infusion in hospital)

Severe dehydration is defined when the child has two or more of the following signs:
Lethargic or unconscious
Sunken eyes
Not able to drink or drinking poorly
Skin pinch goes back very slowly (> 2 seconds)

5) Persistent diarrhea – when severe.

Persistent diarrhea is defined as diarrhea lasting for at least 14 days
When persistent diarrhea is present with some dehydration it is classified as severe persistent diarrhea.

Children with persistent diarrhea that is not severe will be given nutritional therapy; (a) temporarily reduce the amount of animal milk in the diet; (b) provide a sufficient intake of energy, protein, vitamins and minerals to facilitate the repair process in the damaged gut epithelium; (c) avoid giving foods or drinks that may aggravate the diarrhea; and (d) ensure adequate food intake during convalescence to correct any malnutrition. Actively look for and treat intestinal and non-intestinal causes of the diarrhea.

6) Febrile illness with any of these danger signs

Convulsions during the present illness,
Unconscious or lethargic,

Unable to drink or breastfeed,
Vomits everything he or she eats
Stiff neck
Bulging fontanelle

7) Ear infection - mastoiditis

Whenever a child has a tender swelling behind the ear

Visits of enrolled children to the study clinic

These will occur also under the following circumstances.

- whenever the mother brings the child to the clinic because she feels the child needs to see a doctor.
- the child is referred to the clinic by a field worker at a morbidity visit.
- physician requests follow up for an illness. Standard treatment for morbidity based on national guidelines will be prescribed. If a child is diagnosed to have acute lower respiratory infection or clinical pneumonia, the child will be followed at a clinic or home by the physician every 72 hours till the child is well.

Verbal autopsies will be conducted by the physicians for all deaths that occur in enrolled children as early as possible after the child's death.

If an enrolled child is hospitalized, the field worker will record the hospitalization details.

End study activities

At end study (6 months), anthropometric measurements will be taken for all the enrolled children by the field worker, a blood sample by the physician in a sub sample of 256 children (16 blocks) and trained field workers will assess developmental milestones using ASQ-3 on 400 children.

Literature References There is no page limitation on references.

1. Williams BG, Gouws E, Boschi-Pinto C, Bryce J, Dye C. Estimates of world-wide distribution of child deaths from acute respiratory infections. Lancet Infect Dis 2002;2:25-32.
2. Victora CG, Kirkwood BR, Ashworth A, et al. Potential interventions for the prevention of childhood pneumonia in developing countries: improving nutrition. Am J Clin Nutr 1999;70:309-20.
3. Bhandari N, Bahl R, Taneja S, et al. Effect of routine zinc supplementation on pneumonia in children aged 6 months to 3 years: randomised controlled trial in an urban slum. BMJ 2002;324:1358.
4. Brooks WA, Yunus M, Santosham M, et al. Zinc for severe pneumonia in very young children: double-blind placebo-controlled trial. Lancet 2004;363:1683-8.
5. Sazawal S, Black RE, Jalla S, Mazumdar S, Sinha A, Bhan MK. Zinc supplementation reduces the incidence of acute lower respiratory infections in infants and preschool children: a double-blind, controlled trial. Pediatrics 1998;102:1-5.
6. The Zinc Investigators' Collaborative Group. Prevention of diarrhea and pneumonia by zinc supplementation in children in developing countries: pooled analysis of randomized controlled trials. J Pediatr 1999;135:689-97.
7. Dhur A, Galan P, Hercberg S. Folate status and the immune system. Prog Food Nutr Sci 1991;15:43-60.
8. Chandra J, Jain V, Narayan S, et al. Folate and cobalamin deficiency in megaloblastic anemia in children. Indian Pediatr 2002;39:453-7.
9. Chandra S, Chandra RK. Nutrition, immune response, and outcome. Prog Food Nutr Sci 1986;10:1-65.
10. Boles JM, Youinou PY, Garre MA. Single nutrients and nonspecific immunity: role of folic acid on polymorphonuclear leukocytes phagocytosis [letter]. American Journal Of Clinical Nutrition 1982;36:560-1.
11. Youinou P, Garré M, Morin JF, et al. Effect of folic acid deficiency on nonspecific immunity (phagocytic activity and nitroblue - tetrazolium reduction). Pathol Biol (Paris) 1981;29:175-8.
12. Youinou PY, Garre MA, Menez JF, et al. Folic acid deficiency and neutrophil dysfunction. Am J Med 1982;73:652-7.
13. Pinkerton CR, Milla PJ. Methotrexate enterotoxicity: Influence of drug dose and timing in the rat. Br-J-Cancer 1984;49:97-101.
14. Sato E, Ohrui T, Arai H, Sasaki H. Folate deficiency and risk of pneumonia in older people. J Am Geriatr Soc. 2001;49:1739-40.
15. Allen LH. Multiple micronutrients in pregnancy and lactation: an overview. Am J Clin Nutr 2005;81:1206S-12S.
16. Poskitt EM. Infant feeding: a review. Hum Nutr Appl Nutr 1983;37:271-86.
17. Allen LH. B vitamins: proposed fortification levels for complementary foods for young children. J Nutr 2003;133:3000S-7S.
18. Strand TA, Tanjea S, Bhandari N, et al. Folate, but not Cobalamin Status Predicts Respiratory Morbidity in North Indian Children. Submitted 2006.
19. Taneja S, Bhandari N, Strand TA, et al. Cobalamin and Folate Status in Infants and Young Children in a Low Income Community in Delhi. 2006.
20. Bhandari N, Bahl R, Taneja S, et al. Substantial reduction in severe diarrheal morbidity by daily zinc supplementation in young north Indian children. Pediatrics 2002;109:e86.
21. Chanarin I, Deacon R, Lumb M, Muir M, Perry J. Cobalamin-folate interrelations: a critical review. Blood 1985;66:479-89.
22. Parry TE. Cobalamin-folate interrelations [letter]. Blood 1987;69:974-5.
23. Rosenblatt DS, Whitehead VM. Cobalamin and folate deficiency: acquired and hereditary disorders in children. Semin Hematol 1999;36:19-34.
24. Black MM. Micronutrient deficiencies and cognitive functioning. J Nutr 2003;133:3927S-3931S.
25. Bjorke Monsen AL, Ueland PM. Homocysteine and methylmalonic acid in diagnosis and risk assessment from infancy to adolescence. Am J Clin Nutr 2003;78:7-21.
26. Schneede J, Dagnelie PC, van Staveren WA, Vollset SE, Refsum H, Ueland PM. Methylmalonic acid and homocysteine in plasma as indicators of functional cobalamin deficiency in infants on macrobiotic diets. Pediatr Res 1994;36:194-201.
27. Louwman MW, van Dusseldorp M, van de Vijver FJ, et al. Signs of impaired cognitive function in adolescents with marginal cobalamin status. Am J Clin Nutr 2000;72:762-9.
28. Allen LH, Penland JG, Boy E, DeBaessa Y, Rogers LM. Cognitive and neuromotor performance of Guatemalan schoolers with deficient, marginal and normal plasma B-12. FASEB J 1999;13.
29. Siegel EH, Stoltzfus RJ, Kariger PK, et al. Growth indices, anemia, and diet independently predict motor milestone acquisition of infants in south central Nepal. J Nutr 2005;135:2840-4.
30. Holick MF. Vitamin D deficiency. N Engl J Med 2007;357:266-81.

31. Ginde AA, Mansbach JM, Camargo CA. Association between serum 25-hydroxyvitamin D level and upper respiratory tract infection in the Third National Health and Nutrition Examination Survey. Arch Intern Med 2009;169:384-90.
32. Agarwal N, Faridi MMA, Aggarwal A, Singh O. Vitamin D Status of term exclusively breastfed infants and their mothers from India. Acta Paediatr 2010;99:1671-4.
33. Sabetta JR, DePetrillo P, Cipriani RJ, Smardin J, Burns LA, Landry ML. Serum 25-hydroxyvitamin d and the incidence of acute viral respiratory tract infections in healthy adults. PLoS ONE 2010;5:e11088.
34. Tavera-Mendoza LE, White JH. Cell defenses and the sunshine vitamin. Sci Am 2007;297:62-5, 68-70, 72.
35. Sazawal S, Black RE, Ramsan M, et al. Effects of routine prophylactic supplementation with iron and folic acid on admission to hospital and mortality in preschool children in a high malaria transmission setting: community-based, randomised, placebo-controlled trial. Lancet 2006;367:133-43.
36. Ouma P, Parise ME, Hamel MJ, et al. A Randomized Controlled Trial of Folate Supplementation When Treating Malaria in Pregnancy with Sulfadoxine-Pyrimethamine. PLoS Clin Trials 2006;1:e28.
37. English M, Snow RW. Iron and folic acid supplementation and malaria risk. Lancet 2006;367:90-1.
38. Integrated Management of Childhood Illness. Geneva: World Health Organization, 2003.
39. O'Broin S, Kelleher B. Microbiological assay on microtitre plates of folate in serum and red cells. J Clin Pathol 1992;45:344-7.
40. Kelleher BP, Walshe KG, Scott JM, O'Broin SD. Microbiological assay for vitamin B12 with use of a colistin-sulfate-resistant organism. Clin Chem 1987;33:52-4.
41. Moher D, Schulz KF, Altman DG. The CONSORT statement: revised recommendations for improving the quality of reports of parallel-group randomised trials. Lancet 2001;357:1191-4.
