# Supplementary material for: Vitamin B12 and Folic Acid Improve Gross Motor and Problem-Solving Skills in Young North Indian Children: A Randomized Placebo-Controlled Trial
Source: PLoS One. 2015 Jun 22;10(6):e0129915. doi: 10.1371/journal.pone.0129915 (PMC4476750; doi:10.1371/journal.pone.0129915)
Supplement: S2 Protocol — (PDF) [file pone.0129915.s005.pdf]

Protocol Registration Receipt
07/16/2008

Folic Acid and Vitamin B12 in Young Indian Children

This study is not yet open for participant recruitment.

Verified by Centre For International Health, July 2008

Sponsored by:	Centre For International Health<br>Society for Essential Health Action and Training, New Delhi, India<br>Thrasher Research Fund
Information provided by:	Centre For International Health
ClinicalTrials.gov Identifier:	NCT00717730

Purpose

Hypothesis: Supplementation of two recommended daily allowances (RDA) of folic acid with or without simultaneous administration of vitamin B12 reduces the rates of acute lower respiratory tract infections (ALRI), clinical pneumonia and diarrhea.

Design/Methods We will conduct a preventive randomized placebo controlled clinical trial of folic acid and vitamin B12 supplementation in 1000 children aged 6 to 30 months living in a low to middle-income socioeconomic setting in New Delhi, India. Eligible children will be identified through a house-to-house survey. We will include 5 to 10 children every day who will be randomized to 4 treatment groups. These children will be given: 2 RDA of both vitamin B12 and folic acid, 2 RDA of folic acid only, 2 RDA of vitamin B12 only, or placebo. The supplements will be given daily for 6 months.

Condition	Intervention	Phase
Diarrhea<br>Pneumonia	Dietary Supplement: Folic Acid<br>Dietary Supplement: Vitamin B12<br>Dietary Supplement: Placebo folate and B12	Phase 2

Study Type: Interventional
Study Design: Prevention, Factorial Assignment, Double Blind (Subject, Caregiver, Investigator, Outcomes Assessor), Randomized, Efficacy Study
Official Title: Routine Administration of Folic Acid and Vitamin B12 to Prevent Childhood Infections in Young Indian Children

Further study details as provided by Centre For International Health:

Primary Outcome Measure:

- Number of episodes of infection, diarrhea and pneumonia [Time Frame: 6 months] [Designated as safety issue: No]

Secondary Outcome Measures:

- Growth (length for age, weight for age, and length for weight) [Time Frame: 6 month] [Designated as safety issue: No]
- Adverse events (vomiting and gastric discomfort) [Time Frame: 6 months] [Designated as safety issue: Yes]
- Changes in folate, vitamin B12, methyl malonic acid, and homocysteine concentration [Time Frame: 6 months] [Designated as safety issue: No]
- Estimate prevalence of Folate and Vitamin B12 deficiency. [Time Frame: 6 Months] [Designated as safety issue: No]

Estimated Enrollment: 1000
Study Start Date: October 2008
Estimated Study Completion Date: June 2011
Estimated Primary Completion Date: March 2011

Arms	Assigned Interventions
Placebo Comparator: A<br>Placebo dietary supplement	Dietary Supplement: Placebo folate and B12<br>Placebo medicine with no active ingredients
Experimental: B<br>Folic acid, 2 RDA	Dietary Supplement: Folic Acid<br>One RDA of folate corresponds to 75 µg (0.5 x 150) of synthetic folic acid given as a supplement and 2 RDA of folic acid to this group is 150 micrograms per day. In 6 to 11 month old children the RDA for folate is 80 micrograms and the corresponding dose is 160µg .<br>(Food and Nutrition Board, Institute of Medicine, National Academy of Sciences, 2002
Experimental: C<br>Vitamin B12, 2 RDA	Dietary Supplement: Vitamin B12<br>The RDA for vitamin B12 in children 1 - 3 years of age is 0.9 micrograms per day . I.e the daily dose will be 1.8 micrograms. In 6 to 11 month old children the RDA for for B12 is 0.5 micrograms and the daily dose will b 1 micrograms per day. (Food and Nutrition

Arms	Assigned Interventions
	Board, Institute of Medicine, National Academy of Sciences, 2002)
Experimental: D<br>Folic acid and Vitamin B12, 2 RDA	Dietary Supplement: Folic Acid<br>One RDA of folate corresponds to 75 µg (0.5 x 150) of synthetic folic acid given as a supplement and 2 RDA of folic acid to this group is 150 micrograms per day. In 6 to 11 month old children the RDA for folate is 80 micrograms and the corresponding dose is 160µg .<br>(Food and Nutrition Board, Institute of Medicine, National Academy of Sciences, 2002<br>Dietary Supplement: Vitamin B12<br>The RDA for vitamin B12 in children 1 - 3 years of age is 0.9 micrograms per day . I.e the daily dose will be 1.8 micrograms. In 6 to 11 month old children the RDA for for B12 is 0.5 micrograms and the daily dose will b 1 micrograms per day. (Food and Nutrition Board, Institute of Medicine, National Academy of Sciences, 2002)

Pneumonia and diarrhea are among the leading causes of poor health and death in young children of developing countries.

Many of these children have inadequate intakes of several vitamins and minerals. Folate and vitamin B12 are important for normal function of the immune system. Deficiencies of these vitamins are often part of general malnutrition and might be responsible for the excess morbidity and mortality seen in malnourished children. In a recent cohort study in almost 2,500 Indian children we demonstrated that those with poor folate status had higher rates of diarrhea and pneumonia. This study also showed that children that were not breastfed had poor folate status and our analyses suggested that the effect of breastfeeding in preventing respiratory and gastrointestinal infections could be explained by the folate content of breast milk. The finding that poor folate status is related to increased susceptibility to childhood infections needs to be verified in properly conducted clinical trials in populations where folate deficiency is prevalent.

This trial aims to examine whether daily supplementation of two recommended doses of folate or vitamin B12 or both will lessen the incidence of acute lower respiratory tract infections and diarrhea. We will also measure if the supplementation improves the weight and length of supplemented children.

Eligibility

Ages Eligible for Study: 6 Months to 30 Months
Genders Eligible for Study: Both
Accepts healthy volunteers.

Inclusion Criteria:

- Age: 6 to 30 months
- Either sex
- Likely to reside in area for next 6 months
- Availability of informed verbal consent

Exclusion Criteria:

- Severe systemic illness requiring hospitalization
- Severe malnutrition, i.e. weight for height < -3 z of the WHO standard for this age group. For ethical reasons these children require micronutrient supplementation and adequate medical care.
- Lack of consent
- Taking B vitamin supplements that include folic acid and vitamin B12.
- Severe anemia (Hb < 7 g/dL). This would be a temporary exclusion and the children will be enrolled if this is successfully treated.
- Ongoing acute infection with fever or infection that requires medical treatment. This would be a temporary exclusion and the children will be enrolled after recovery.

Contacts and Locations

Contacts

Sunita Taneja, MBBS, PhD	00 91 9811206456	community.research@cih.uib.no
Nita Bhandari, MBBS, PhD	00 91 11 46080629	community.research@cih.uib.no

Locations

India, Delhi

Society for Essential Health Action and Training — Not yet recruiting

New Delhi, Delhi, India

Contact: Sunita Taneja, MBBS, PhD 00 91 9811206456

Contact: Nita Bhandari, MBBS, PhD 00 91 11 46080629 community.research@cih.uib.no

Investigators

Principal Investigator:	Tor A Strand, MD, PhD	University of Bergen
Principal Investigator:	Sunita Taneja, MBBS, PhD	Society for Essential Health Action and Training
Study Director:	Nita Bhandari, MBBS, PhD	Society for Essential Health Action and Training

More Information

Publications:

Taneja S, Bhandari N, Strand TA, Sommerfelt H, Refsum H, Ueland PM, Schneede J, Bahl R, Bhan MK. Cobalamin and folate status in infants and young children in a low-to-middle income community in India. Am J Clin Nutr. 2007 Nov;86(5):1302-9.

Strand TA, Taneja S, Bhandari N, Refsum H, Ueland PM, Gjessing HK, Bahl R, Schneede J, Bhan MK, Sommerfelt H. Folate, but not vitamin B-12 status, predicts respiratory morbidity in north Indian children. Am J Clin Nutr. 2007 Jul;86(1):139-44.

Allen LH. Multiple micronutrients in pregnancy and lactation: an overview. Am J Clin Nutr. 2005 May;81(5):1206S-1212S. Review.

Smith AD, Kim YI, Refsum H. Is folic acid good for everyone? Am J Clin Nutr. 2008 Mar;87(3):517-33.

Sazawal S, Dhingra U, Dhingra P, Hiremath G, Kumar J, Sarkar A, Menon VP, Black RE. Effects of fortified milk on morbidity in young children in north India: community based, randomised, double masked placebo controlled trial. BMJ. 2007 Jan 20;334(7585):140. Epub 2006 Nov 28.

Tielsch JM, Khatry SK, Stoltzfus RJ, Katz J, LeClerq SC, Adhikari R, Mullany LC, Shresta S, Black RE. Effect of routine prophylactic supplementation with iron and folic acid on preschool child mortality in southern Nepal: community-based, cluster-randomised, placebo-controlled trial. Lancet. 2006 Jan 14;367(9505):144-52.

Fawzi WW, Msamanga GI, Spiegelman D, Urassa EJ, McGrath N, Mwakagile D, Antelman G, Mbise R, Herrera G, Kapiga S, Willett W, Hunter DJ. Randomised trial of effects of vitamin supplements on pregnancy outcomes and T cell counts in HIV-1-infected women in Tanzania. Lancet. 1998 May 16;351(9114):1477-82.

Sazawal S, Black RE, Ramsan M, Chwaya HM, Stoltzfus RJ, Dutta A, Dhingra U, Kabole I, Deb S, Othman MK, Kabole FM. Effects of routine prophylactic supplementation with iron and folic acid on admission to hospital and mortality in preschool children in a high malaria transmission setting: community-based, randomised, placebo-controlled trial. Lancet. 2006 Jan 14;367(9505):133-43. Erratum in: Lancet. 2006 Jan 28;367(9507):302.

Fawzi WW, Msamanga GI, Urassa W, Hertzmark E, Petraro P, Willett WC, Spiegelman D. Vitamins and perinatal outcomes among HIV-negative women in Tanzania. N Engl J Med. 2007 Apr 5;356(14):1423-31.

Tamura T, Yoshimura Y, Arakawa T. Human milk folate and folate status in lactating mothers and their infants. Am J Clin Nutr. 1980 Feb;33(2):193-7.

Khambalia A, Latulippe ME, Campos C, Merlos C, Villalpando S, Picciano MF, O'connor DL. Milk folate secretion is not impaired during iron deficiency in humans. J Nutr. 2006 Oct;136(10):2617-24.

Responsible Party:	University of Bergen (Tor A Strand/ Researcher)
Study ID Numbers:	RCN172226
Health Authority:	Norway: The National Committees for Research Ethics in Norway
